# Increases in activity of proteasome and papain-like cysteine protease in Arabidopsis autophagy mutants: back-up compensatory effect or cell-death promoting effect?

**DOI:** 10.1093/jxb/erx482

**Published:** 2018-01-27

**Authors:** Marien Havé, Thierry Balliau, Betty Cottyn-Boitte, Emeline Dérond, Gwendal Cueff, Fabienne Soulay, Aurélia Lornac, Pavel Reichman, Nico Dissmeyer, Jean-Christophe Avice, Patrick Gallois, Loïc Rajjou, Michel Zivy, Céline Masclaux-Daubresse

**Affiliations:** 1 INRA-AgroParisTech, Institut Jean-Pierre Bourgin, France; 2 UMR GQE- le Moulon, INRA, Université Paris-Sud, CNRS, AgroParisTech, Université Paris-Saclay, France; 3 UCBN, INRA, UMR INRA-UBCN 950 Ecophysiologie Végétale, Agronomie & Nutrition N.C.S., Université de Caen Normandie, France; 4 Independent Junior Research Group on Protein Recognition and Degradation, Leibniz Institute of Plant Biochemistry (IPB), Weinberg 3, Halle (Saale), Germany and Science Campus Halle – Plant-based Bioeconomy, Germany; 5 School of Biological Sciences, Faculty of Biology, Medicine and Health, University of Manchester, UK; 6 Swedish University of Agricultural Sciences, Sweden

**Keywords:** AALP, metacaspase, nitrogen remobilization, senescence

## Abstract

Autophagy is essential for protein degradation, nutrient recycling, and nitrogen remobilization. Autophagy is induced during leaf ageing and in response to nitrogen starvation, and is known to play a fundamental role in nutrient recycling for remobilization and seed filling. Accordingly, ageing leaves of Arabidopsis autophagy mutants (*atg*) have been shown to over-accumulate proteins and peptides, possibly because of a reduced protein degradation capacity. Surprisingly, *atg* leaves also displayed higher protease activities. The work reported here aimed at identifying the nature of the proteases and protease activities that accumulated differentially (higher or lower) in the *atg* mutants. Protease identification was performed using shotgun LC-MS/MS proteome analyses and activity-based protein profiling (ABPP). The results showed that the chloroplast FTSH (FILAMENTATION TEMPERATURE SENSITIVE H) and DEG (DEGRADATION OF PERIPLASMIC PROTEINS) proteases and several extracellular serine proteases [subtilases (SBTs) and serine carboxypeptidase-like (SCPL) proteases] were less abundant in *atg5* mutants. By contrast, proteasome-related proteins and cytosolic or vacuole cysteine proteases were more abundant in *atg5* mutants. Rubisco degradation assays and ABPP showed that the activities of proteasome and papain-like cysteine protease were increased in *atg5* mutants. Whether these proteases play a back-up role in nutrient recycling and remobilization in *atg* mutants or act to promote cell death is discussed in relation to their accumulation patterns in the *atg5* mutant compared with the salicylic acid-depleted *atg5/sid2* double-mutant, and in low nitrate compared with high nitrate conditions. Several of the proteins identified are indeed known as senescence- and stress-related proteases or as spontaneous cell-death triggering factors.

## Introduction

Autophagy is a universal mechanism that facilitates the degradation of unwanted cell constituents in the lytic compartments of eukaryotic cells. Autophagy is essential for the recycling of cellular material and controls nitrogen remobilization and grain-filling in Arabidopsis (Guiboileau *et al.*, 2012; Masclaux-Daubresse *et al.*, 2017). The autophagy machinery consists of the formation of cytosolic double-membrane vesicles, termed autophagosomes, that engulf and sequester unwanted cytoplasmic constituents such as damaged organelles and protein aggregates (Liu and Bassham, 2012). Degradation does not occur directly inside the autophagosome as molecules destined for recycling are transported to the lytic vacuoles where proteases and hydrolases operate. Autophagosome formation involves the products of *AUTOPHAGY* (*ATG*) genes, previously discovered in yeast by the pioneering work of Professor Yoshinori Ohsumi (Nobel Prize in medicine or physiology 2016; Tsukada and Ohsumi, 1993). Subsequently, homologous genes in plants and animals have been found for almost all *ATG*s. Among the 50 *ATG* genes discovered in yeast, 18 are part of the autophagy core machinery (Yang and Bassham, 2015). These genes are absolutely essential to the formation of autophagosomes. In Arabidopsis, the core machinery genes are either single or belong to gene families. In particular, *ATG5*, involved in the ATG5–ATG12 conjugation system, is essential for the formation of the ATG8-PE (PE, phosphatidylethanolamine) conjugate that features on the autophagosome membrane and helps the expansion of the vesicle membrane (Masclaux-Daubresse *et al.*, 2017). The *atg5* Arabidopsis mutants have been intensively studied (Thompson *et al.*, 2005; Yoshimoto *et al.*, 2009; Lenz *et al.*, 2011; Guiboileau *et al.*, 2012, 2013; Masclaux-Daubresse *et al.*, 2014; Floyd *et al.*, 2015; Wang *et al.*, 2015; Fahy *et al.*, 2017). Like the other *atg* mutants studied so far (*atg9*, *atg7*, and *atg2*), the *atg5* mutants present smaller rosettes, hypersensitivity to N and C starvation, and reduced yield. Using ^15^N-tracer experiments, Guiboileau *et al.* (2012) showed that the *atg5* and *atg9* mutants and the *atg18a*-RNAi line are strongly affected in N remobilization, and we have verified further that this is also the case for many other *atg* single-mutants (C. Masclaux-Daubresse, unpublished results). Several lines of evidence show that N and C leaf metabolisms are strongly affected in *atg5*, *atg9*, and *atg18a*-RNAi (Guiboileau *et al.*, 2013; Masclaux-Daubresse *et al.*, 2014). Mutants have lower starch, sucrose, and hexose contents in their leaves and higher amounts of amino acids (especially glutamate, aspartate, and methionine). They also display higher protein and RNA concentrations relative to wild-type plants. In order to try and explain such phenotypes, Guiboileau *et al.* (2013) measured the endo-, carboxy-, and amino-peptidase activities, and a series of western blots were also carried out in order to determine whether protein accumulation was selective. The results showed that several proteins and peptides were specifically accumulated in the leaves of *atg* mutants. The absence of transcriptional changes suggested that these proteins and peptides were specific cargoes. Surprisingly, protease activities were significantly increased in the leaves of *atg* mutants (Guiboileau *et al.*, 2013), showing that the accumulation of the peptides and proteins over-abundant in the *atg* rosettes was not related to lower proteolysis capacity. In was then hypothesized that in the absence of autophagosome trafficking, vacuole proteases and their cytoplasmic substrates cannot co-localize, resulting in protein over-accumulation.

The aim of this study was to identify which protease activities are increased in autophagy mutants in order to compensate for the defects in autophagy-dependent protein recycling and to provide alternative remobilization pathways. Proteomics shotgun LC-MS/MS analyses were carried out in order to identify the proteases and proteins with increased or decreased accumulation in *atg5*. Because part of the phenotypes of autophagy mutants is related to the increase of salicylic acid (SA) in *atg* leaves, the *atg5/sid2* double-mutant was also analysed in order to discriminate SA-dependent and SA-independent changes. As a low-nitrate regime is known to promote nitrogen remobilization and autophagy activity, mutants were grown under both low- and high-nitrate conditions. Low-nitrate is indeed more favourable for the detection of autophagy-related phenotypes (Guiboileau *et al.*, 2012, 2013; Masclaux-Daubresse *et al.*, 2014). In addition to the identification of the proteases differentially accumulated in *atg* mutants, we monitored whether they were active by using specific probes (Morimoto and van der Hoorn, 2016), and performed western blots in order to evaluate the respective amounts of their pro- and active-forms. The results are discussed with regards to the known relationships that exist between protease activities, leaf senescence, N remobilization, and cell death. This work reveals new candidates that may participate in the management of N remobilization and possibly in the last steps of autophagy in Arabidopsis leaves.

## Material and methods

### Plant material and growth conditions

*Arabidopsis thaliana* (L.) Columbia wild-type, *atg5* (SALK_020601), *atg5/sid2*, and *sid2* mutants have been previously characterized by Yoshimoto *et al.* (2009) and Guiboileau *et al.* (2012, 2013). Plants were cultivated according to Guiboileau *et al.* (2013) under low- (LN; 2 mM) and high- (HN; 10 mM) nitrate conditions. Adequate growth conditions for the testing of nitrogen-limitation effects on Arabidopsis plants have previously been established by Loudet *et al.* (2003*a*, 2003*b*) and further improved by Lemaître *et al.* (2008), (see Supplementary Method S1 at *JXB* online for details). Whole rosettes were harvested at 60 d after sowing (DAS). Three independent plant replicates were sampled. For HN, each biological replicate contained four rosettes; for the LN, each biological replicate contained 24 rosettes. Harvests commenced 2 h into the 8-h photoperiod and were completed within 1 h. Samples were stored at –80 °C for subsequent use. Three batches of plants were cultured, providing samples from three independent experiments. The analyses detailed below were then performed on three biological replicates and repeated on samples from 2–3 independent batches of plants. For validation of the anti-RD21A antibody, we used the homozygous *rd21a* SALK_090550C T-DNA insertion mutant (Wang *et al.*, 2008). For validation of the anti-CATHB3 antibody, the SALK_19630 *cathb3* homozygous T-DNA insertion mutant was used (Ge *et al.*, 2016).

### Shotgun proteomic analysis

Leaf total proteins were extracted, digested, and analysed by LC-MS/MS using the PAPSSO platform (INRA, Le Moulon, Gif sur Yvette; see Supplementary Method S2 for details). Protein identification was performed using X! Tandem Piledriver (version 2015.04.01; http://www.thegpm.org/TANDEM/index.html) by querying the MS/MS data against the TAIR10 protein library together with a custom contaminant database (trypsin, keratins). Identified proteins were filtered and grouped using X! Tandem Pipeline (3.4.1; pappso.inra.fr/bioinfo/xtandempipeline/) (Langella *et al.*, 2017) according to two criteria: (1) a minimum of two different peptides required with an *E*-value smaller than 0.01, and (2) a protein *E*-value (calculated as the product of unique peptide *E*-values) smaller than 10^−5^. The false discovery rates (FDRs) at the peptide and protein levels were 0.03% and 0.0%, respectively. Relative peptide quantification by peak-area integration on eXtracted ion chromatogram (XICs) was performed using the MassChroQ software (pappso.inra.fr/bioinfo/masschroq/) (Valot *et al.*, 2011). Relative protein abundance was calculated and defined as the sum of peptide intensities considering only (1) reproducible peptides, (2) specific peptides, and (3) correlated peptides belonging to the same protein (see Supplementary Method S2 for details). When the peptides of a protein were not present or not reproducibly observed in one or more conditions, spectral counting (SC) was used in place of XICs analysis.

### Activity-based protein profiling of papain-like cysteine proteases, proteasome and serine proteases

Papain-like cysteine proteases (PLCPs), serine proteases, and proteasome activity profiling were assayed using DCG-04 (van der Hoorn *et al.*, 2004), desthiobiotin-FP (DFP; Thermo Scientific) and MVB072 (Kolodziejek *et al.*, 2011), respectively. DCG-04 was synthesized as described by Greenbaum *et al.* (2000). The protein extract obtained by homogenizing 200 mg of frozen material with 450 µl of water (1.5% polyvinylpolypyrrolidone, w/v) was centrifuged (20 000 *g*, 15 min, 4 °C). Protein concentration was determined using the 2D Quant kit (SIGMA-ALDRICH). All labelling reactions were conducted in 100 µl volume containing 100 µg of proteins in the dark at room temperature. For PLCPs, proteins were labelled with 50 mM sodium acetate (NaAc), pH 5.5, 2 mM DTT, and 5 µM DCG-04 for 150 min. For serine proteases, proteins were labelled with 50 mM NaAc, pH 5.5, or 50 mM Hepes-KOH, pH 7.5, and 2 µM desthiobiotin-FP for 1 h. For the proteasome, proteins were labelled with 50 mM Hepes-KOH, pH 7.5, and 1 µM MVB072 for 1 h. For each genotype, an additional sample corresponding to a mixture of an equal amount of soluble proteins from high-N and low-N was used as a control to check for probe specificity. Control samples were labelled with each probe as already described and were also subjected to competition assays consisting of a pre-treatment with 100 µM E-64, 50 µM DFP, or 50 µM epoxomicin for 30 min before adding DCG-04, desthiobiotin-FP, or MVB072, respectively. As a no-probe-control (NPC), control samples were incubated with an equal volume of probe solvent (DMSO). Labelling reactions were stopped by addition of 25 µl of 4× SDS-PAGE loading buffer and β-mercaptoethanol (355 mM) and heated at 90 °C for 10 min. For each sample, 10 µg of labelled proteins were separated by 12% or 15% SDS-PAGE. Resolved MVB072-labeled proteins were visualized by in-gel fluorescence scanning using a Typhoon 9400 scanner (GE Healthcare Life Science) with excitation and emission wavelengths at 532 and 580 nm, respectively. Resolved DCG-04- and desthiobiotin-FP-labelled proteins were transferred to a polyvinylidene fluoride membrane. Membranes were incubated overnight in 1% BSA (w/v) in PBS-T 0.1% and incubated with streptavidin-HRP (Ultrasensitive) for 1 h. The chemiluminescence signal was visualized using an ImageQuant LAS 4000 (GE Healthcare Life Science). Colloidal Blue-stained gels were used to monitor protein loading. Fluorescence, chemiluminescence, and Coomassie signals were quantified using the ImageQuant software (GE Healthcare Life Science).

### Affinity purification and identification of PLCPs

Only plants grown under low-N were analysed. For each genotype, soluble proteins from four independent rosette bulk samples were extracted as described above. An equal amount of proteins (750 µg) from first and second, and from third and fourth bulk samples were mixed to obtain two mixed protein solutions per genotype. Then, 1.5 mg of proteins were labelled with 50 mM NaAc, pH 5.5, 2 mM DTT, and 5 µM DCG-04 in 1 ml of reaction volume and incubated for 150 min at room temperature in the dark. Labelling reactions were stopped and the biotinylated proteins were purified as described by Chandrasekar *et al.* (2014), except that avidin beads were used (SIGMA-ALDRICH). The elution step was repeated once and the two eluates were mixed and separated by 12% SDS-PAGE. Gels were stained with silver nitrate and bands with a molecular mass between 25 and 40 kDa, corresponding to bands observed in the activity-based protein profiling (ABPP) experiment, were excised with a OneTouch GridCutter (2.0 × 7.0 mm; Gel Company). Each slice was placed into a 0.5-ml tube, digested, and analysed by LC-MS/MS as detailed in Supplementary Method S3. Proteomics data analyses were performed as explained in Supplementary Method S3. Identified proteins were filtered and grouped using X! Tandem Pipeline (3.4.2) according to the following criteria: (1) a minimum of two different peptides required with an *E*-value smaller than 0.05, and (2) a protein *E*-value smaller than 10^−4^.

### Western blots

Total proteins were extracted by homogenizing 100 mg of frozen material in 500 µl of buffer [7 M urea, 2 M thiourea, 30 mM Tris-HCl pH 8.8, 4% CHAPS (w/v), 0.2% triton X100 (w/v), 20 mM DTT, and 1× cOmplete protease inhibitor cocktail (Roche)]. Extracts were centrifuged and protein concentration was determined as described above. For each sample, 5 µg of proteins were separated on 12% SDS-PAGE. Resolved proteins were electroblotted (Trans-Blot Turbo transfer system; Bio-Rad). Polypeptide detections were performed using antibodies raised against the RD21A-specific peptide DELPESIDWRKKG, against SAG12 (Agrisera, Vännäs, Sweeden), and against the N-terminal end of the CATHB3 mature protein (synthetic peptide LPKAFDARTAWPQC). Primary antibodies were diluted 1:1000 in 5% milk PBS-T 0.1% buffer for membrane incubation. Secondary HRP peroxidase antibodies (1:10000) were used for chemiluminescence detection. Signals were detected using an ImageQuant LAS 4000 (GE Healthcare Life Science). Coomassie Brilliant Blue-stained gels were used to verify that an equal amount of protein was loaded in each lane. RD21A and CATHB3 antibody specificities were tested using *rd21A* and *cathb3* mutants as negative controls (see Supplementary Fig. S1).

### Bioinformatics and statistical analyses

All statistical analyses were performed using the R software (https://www.r-project.org/). Data were analysed using two-way ANOVA with genotype and nutrition conditions as variable factors, followed by a Tukey’s *post-hoc* test. For the relative quantification of proteins by the XICs method, statistical analyses were carried out on log_10_-transformed protein abundance. Proteins corresponding to proteases with a Tukey’s *P*-value ≤0.05 were considered to be significantly differentially accumulated. Fold-change (FC) ratios were determined as abundance ratios of *atg5* versus Col and of *atg5/sid2* versus *sid2*. No FC threshold was applied as even a small change in protease abundance can have a strong impact on the proteome due to post-translational regulation and the large substrate spectrum of proteases. In order to identify proteases among the proteins detected by LC-MS/MS, Gene Ontology and functional information for each protein were analysed using the following databases: MEROPS (https://www.ebi.ac.uk/merops/), UniProt (www.uniprot.org/), agriGO (bioinfo.cau.edu.cn/agriGO), and Mapman (mapman.gabipd.org/).

## Accession numbers

AT4G38220; AT2G27020; AT2G24200; AT4G20850; AT5G35590; AT4G31300; AT3G22110; AT2G05840; AT4G14800; AT3G60820; AT1G53750; AT3G05530; AT4G17510; AT5G05780; AT5G58290; AT5G10540; AT1G21720; AT1G56450; AT5G42790; AT4G01610; AT1G47128; AT1G53850; AT5G45890; AT5G51070; AT3G13235; AT1G79340; AT1G50380; AT3G51260; AT5G23540; AT4G38630; AT1G51710; AT5G66140; AT4G30910; AT4G39090; AT5G43060; AT5G10760; AT1G16470; AT1G79210; AT2G14260; AT5G23140; AT4G16190; AT5G26860; AT3G14290; AT3G20630; AT2G41790; AT4G36760; AT5G36210; AT1G50250; AT5G42270; AT4G36195; AT1G09750; AT3G54400; AT3G02110; AT2G39850; AT1G52510; AT2G47940; AT2G35780; AT5G39830; AT3G27925; AT3G14067; AT3G19170; AT5G42390; AT3G24590; AT1G13270; AT5G05740; AT2G30950; AT4G18370; AT3G52500; AT5G42240; AT1G06430; AT5G23210; AT3G05350; AT5G65760; AT1G67700; AT3G61820; AT1G01300; AT4G34980; AT2G05920; AT3G18490; AT2G33530; AT4G21650; AT5G08260; AT1G09130; AT1G21750; AT1G47710; AT4G16500; AT517290; AT5G60360; AT5G50260; AT1G02305; AT4G35350.

## Results

### LC-MS/MS identification of protease proteins in autophagy mutants

**Fig. 1.**
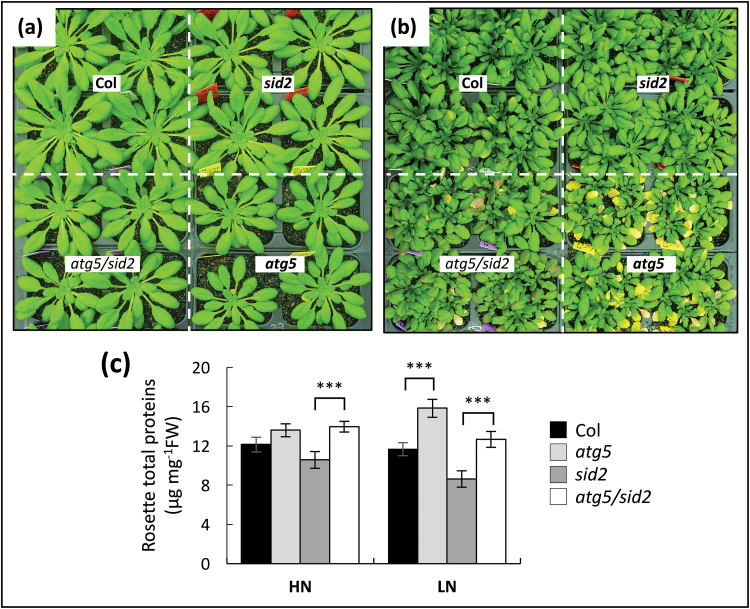
Autophagy-defective mutants accumulate proteins in their rosette leaves. Plants were cultivated under high- (a) or low- (b) nitrate conditions for 60 d. Total proteins (c) were extracted from rosette leaves using TCA/acetone and suspended in denaturing buffer. Values are means ±SD of three biological replicates. Significant differences from controls (i.e. *atg5* versus Col, and *atg5/sid2* versus *sid2*) are indicated: ***P<0.001 (ANOVA and Tukey’s *post-hoc* test for multiple comparisons). Three independent plant cultures showed similar results: only one representative sample is presented here.

Shotgun LC-MS/MS proteome analyses were carried out to identify the nature of the proteases specifically accumulated in *atg5* mutants. Total proteins were extracted from the autophagy-defective lines (*atg5* and *atg5/sid2*) and their respective controls (Col and *sid2*) grown for 60 d under high-N (HN) and low-N (LN) as previously described by Guiboileau *et al.* (2013). The *atg5/sid2* double-mutant was analysed in addition to *atg5* in order to discriminate against the side effects of salicylic acid (SA) (Guiboileau *et al.*, 2012). With regards to previous studies (Yoshimoto *et al.*, 2009; Guiboileau *et al.*, 2012, 2013; Masclaux-Daubresse *et al.*, 2014), we preferred here to consider *atg5/sid2* versus *sid2* rather than *atg5/*NahG versus NahG in order to discriminate against SA effects because of the lower SA content found in the *sid2* background relative to the NahG background (see Supplementary Fig. S2). The rosette phenotypes at harvest are shown in Fig. 1a, b. Under HN conditions, no leaf senescence phenotype was observed in any of the four genotypes (Fig.1a). Under LN conditions, a slight senescence phenotype was observed on the oldest leaves of *atg5*; this phenotype was mitigated in *atg5/sid2*. In accordance with Guiboileau *et al.* (2013), we confirmed that the total protein concentration was higher in *atg5* relative to the Col wild-type under LN, and significantly higher in *atg5/sid2* compared to *sid2* under both LN and HN (Fig.1c).

LC-MS/MS shotgun proteome analyses were then carried out on the total protein extracted from three independent biological repeats. XICs analysis permitted the relative quantification of proteins when peptides were detected in all the genotypes. When a peptide was undetectable in one of the two genotypes under comparison, individual peptide quantification by spectral count (SC) was performed. XICs and SC analyses, respectively, identified increased accumulation of 47 and decreased accumulation of 36 protease-related proteins in the autophagy-defective lines relative to control lines (Tables 1 and 2).

**Table 1.** List of the proteases with significantly increased accumulation in *atg5* versus Col, and in *atg5/sid2* versus *sid2*

	M ^ (1) ^	Accession (TAIR10)	Name ^ (2) ^	Catalytic class ^ (3) ^	Predicted subcellular localization ^ (4) ^	Fold-change ^ (5) ^			
						*atg5*		*atg5/sid2*	
						HN	**LN**	**HN**	**LN**
Class 1	XICs	AT4G38220	AQI	Met	ER	**1.63**	**1.77**	**1.84**	**2.21**
	XICs	AT2G27020	PAG1	Thr	20S (CP α)	**1.41**	**1.79**	**1.64**	**1.84**
	XICs	AT2G24200	LAP1	Met	PM,C	**1.44**	**1.55**	**1.42**	**1.41**
	XICs	AT4G20850	TPP2	Ser	Pl	**1.38**	**1.48**	**1.38**	**1.55**
	XICs	AT5G35590	PAA1	Thr	20S (CP α)	**1.24**	**1.58**	**1.28**	**1.63**
	XICs	AT4G31300	PBA1	Thr	20S (CP β)	**1.33**	**1.37**	**1.40**	**1.41**
	XICs	AT3G22110	PAC1	Thr	20S (CP α)	**1.28**	**1.51**	**1.20**	**1.46**
Class 2	XICs	AT2G05840	PAA2	Thr	20S (CP α)	1.28	**1.73**	**1.43**	**1.72**
	XICs	AT4G14800	PBD2	Thr	20S (CP β)	1.19	**1.80**	**1.43**	**1.65**
	XICs	AT3G60820	PBF1	Thr	20S (CP β)	1.15	**1.49**	**1.23**	**1.52**
	XICs	AT1G53750	RPT1A	non-catalytic	19S (RP base)	1.25	**1.42**	**1.29**	**1.47**
Class 3	XICs	AT3G05530	RPT5A	non-catalytic	19S (RP base)	1.31	**2.29**	1.39	**2.33**
	XICs	AT4G17510	UCH3	Cys	C	1.55	**2.27**	1.82	**2.09**
	XICs	AT5G05780	RPN8A	non-catalytic	19S (RP lid)	1.18	**2.44**	1.31	**1.88**
	XICs	AT5G58290	RPT3	non-catalytic	19S (RP base)	1.08	**2.17**	1.38	**2.13**
	XICs	AT5G10540	TOP2	Met	C,Pl	1.05	**2.26**	1.44	**2.03**
	XICs	AT1G21720	PBC1	Thr	20S (CP β)	1.33	**1.96**	1.41	**2.01**
	XICs	AT1G56450	PBG1	Thr	20S (CP β)	1.18	**1.86**	1.21	**1.78**
	XICs	AT5G42790	PAF1	Thr	20S (CP α)	1.41	**1.80**	1.14	**1.83**
	XICs	AT4G01610	CATHB3	Cys	E,V	1.13	**1.84**	0.85	**1.79**
	XICs	AT1G47128	RD21A	Cys	E,V	0.91	**1.46**	0.74	**1.57**
	SC	AT1G53850	PAE1	Thr	20S (CP α)	1.75	**1.69**	1.50	**1.67**
	SC	AT5G45890	SAG12	Cys	V	1.50 (+)	**22.50 (+**)	1.00 (+)	**24.50 (+**)
Class 4	XICs	AT5G51070	CLPD	Ser	Pl	**1.49 (+**)	**2.37 (+**)	1.13	1.40 (+)
	XICs	AT3G13235	DDI1	Asp	C	**1.53**	**1.61**	1.21	1.21
	XICs	AT1G79340	AMC4	Cys	PM,C	**1.41**	**1.51**	1.28	1.36
	XICs	AT1G50380	_	Ser	C	**1.30**	**1.34**	1.13	1.08
Class 5	XICs	AT3G51260	PAD1	Thr	20S (CP α)	1.58	**2.53**	1.72	1.79
	XICs	AT5G23540	RPN11	non-catalytic	19S (RP lid)	1.10	**1.34**	1.10	1.26
	XICs	AT4G38630	RPN10	non-catalytic	19S (RP lid)	1.19	**1.31**	1.28	1.28
	XICs	AT1G51710	UBP6	Cys	C	1.13	**1.22**	1.06	1.05
	SC	AT5G66140	PAD2	Thr	20S (CP α)	1.25	**5.00**	2.33	1.67
	SC	AT4G30910	LAP3	Met	Pl	0.89	**1.35**	1.20	1.45
	XICs	AT4G39090	RD19A	Cys	V,N	1.15	**1.74**	0.96	1.56
	XICs	AT5G43060	RD21B	Cys	E,V	0.99 (+)	**1.49 (+**)	0.59	1.36
	SC	AT5G10760	AED1	Asp	E	1.80 (+)	**3.33 (+**)	0.67	1.50 (+)
Class 6	SC	AT1G16470	PAB1	Thr	20S (CP α)	1.50	1.40	**2.22**	1.35
	SC	AT1G79210	PAB2	Thr	20S (CP α)	1.55	1.11	**2.00**	1.21
	XICs	AT2G14260	PIP	Ser	C,Pl	1.18	1.23	**1.48**	1.24
Class 7	XICs	AT5G23140	CLPP2	Ser	M	0.93	2.42	0.86	**2.77**
	XICs	AT4G16190	RD19C	Cys	E,V	1.05	1.46	1.18	**2.08**
	XICs	AT5G26860	LON1	Ser	M	1.21	1.31	1.02	**1.40**
	SC	AT3G14290	PAE2	Thr	20S (CP α)	1.40	1.33	1.42	**1.63**
Class 8	XICs	AT3G20630	UBP14	Cys	C	**1.43**	1.40	**1.42**	1.31
Class 9	XICs	AT2G41790	PXM16	Met	Pe	**1.54**	1.29	1.43	1.11
Class 10	XICs	AT4G36760	APP1	Met	PM,C	**1.51**	**1.72**	1.27	**1.62**
Class 11	XICs	AT5G36210	_	Ser	Pl	**1.36**	1.24	**1.57**	**1.31**

Plants were grown under high- (HN) or low- (LN) nitrate conditions. ^(1)^ XICs or Spectral counting (SC) methods. ^(2)^ Protein names according to UniProt and TAIR. ^(3)^ Catalytic classes according to MEROPS: asp, aspartic proteases; cys, cysteine proteases; met, metallo-proteases; ser, serine proteases; thr, threonine proteases; non-catalytic, non-catalytic proteasome regulatory sub-units. ^(4)^ Predicted subcellular localizations according to SUBA3 (SUBAcon), Bio-Analytic Ressource for Plant Biology (Cell eFP Viewer) and Marshall *et al.* (2015). 20S (CP α), proteasome 20S core protease α; 20S (CP β), proteasome 20S core protease β; 19S (RP base), proteasome 19S regulatory particle base; 19S (RP lid), proteasome 19S regulatory particle lid; C, cytosol; ER, endoplasmic reticulum; E, extracellular; M, mitochondrion; Pe, peroxisome; PM, plasma membrane; Pl, plastid; V, vacuole. ^(5)^ Fold-change ratios were calculated by dividing protein abundance in *atg5* and *atg5/sid2* by protein abundance in Col and in *sid2*, respectively. Entries in bold represent significantly increased accumulations, all other entries are non-significant changes (*n* = 3; *P*<0.05; ANOVA and Tukey’s *post-hoc* test for multiple comparisons). (+) significant increase in gene expression (Log_2_ fold-change of mRNA levels >1.2 with *P*-value <10^–5^, rank product and FDR estimation).

**Table 2.** List of the proteases with significantly decreased accumulation in *atg5* versus Col, and *atg5/sid2* versus *sid2*

	M ^ (1) ^	Accession (TAIR10)	Name ^ (2) ^	Catalytic class ^ (3) ^	Predicted subcellular localization ^ (4) ^	Fold-change ^ (5) ^			
						*atg5*		*atg5/sid2*	
						HN	**LN**	**HN**	**LN**
Class 1	XICs	AT1G50250	FTSH1	met	Pl	**0.76**	**0.49**	**0.67**	**0.42**
	XICs	AT5G42270	FTSH5	met	Pl	**0.71**	**0.51**	**0.67**	**0.48**
	XICs	AT4G36195	_	ser	V,PM	**0.66**	**0.61**	**0.77**	**0.52**
Class 2	XICs	AT1G09750	AED3	asp	E	0.78	**0.63**	**0.65**	**0.61**
	XICs	AT3G54400	_	asp	E,Pl	0.77	**0.62**	**0.72**	**0.59**
Class 3	XICs	AT3G02110	SCPL25	ser	E	**0.60**	**0.46**	0.70	**0.44**
	XICs	AT2G39850	SBT4.1	ser	E	**0.60**	**0.48**	0.78	**0.59**
	XICs	AT1G52510	_	Ser	Pl	**0.69**	**0.52**	0.81	**0.54**
	XICs	AT2G47940	DEGP2	Ser	Pl	**0.71**	**0.56**	0.81	**0.58**
	XICs	AT2G35780	SCPL26	Ser	V,E	**0.81**	**0.58**	0.74	**0.46**
	XICs	AT5G39830	DEGP8	Ser	Pl	**0.66**	**0.57**	0.75	**0.67**
	XICs	AT3G27925	DEGP1	Ser	Pl	**0.76**	**0.68**	0.77	**0.68**
	XICs	AT3G14067	SASP	Ser	E	**0.78**	**0.73**	0.88	**0.74**
	XICs	AT3G19170	PREP1	met	Pl	**0.81**	**0.74**	0.84	**0.74**
Class 4	XICs	AT5G42390	SPP	met	Pl	0.81	**0.38**	0.75	**0.43**
	XICs	AT3G24590	PLSP1	Ser	Pl	0.69	**0.36**	0.75	**0.50**
	XICs	AT1G13270	MAP1C	met	Pl	0.70	**0.56**	0.71	**0.48**
	XICs	AT5G05740	EGY2	met	Pl	0.78	**0.55**	0.85	**0.56**
	XICs	AT2G30950	FTSH2	met	Pl	0.75	**0.57**	0.68	**0.54**
	XICs	AT4G18370	DEGP5	Ser	Pl	0.66	**0.54**	0.83	**0.65**
	XICs	AT3G52500	_	Asp	E	0.82	**0.65**	0.77	**0.61**
	XICs	AT5G42240	SCPL42	Ser	E	0.86	**0.66**	0.87	**0.62**
	XICs	AT1G06430	FTSH8	met	Pl	0.77	**0.71**	0.74	**0.58**
	XICs	AT5G23210	SCPL34	Ser	E	0.86	**0.65**	0.87	**0.68**
	XICs	AT3G05350	_	met	Pl	0.83	**0.66**	0.88	**0.69**
	XICs	AT5G65760	_	Ser	Pl,V	0.86	**0.70**	0.93	**0.68**
Class 5	XICs	AT1G67700	HHL1	met	Pl	0.76	**0.39**	0.74	0.53
	XICs	AT3G61820	_	Asp	E	0.84	**0.61**	0.72	0.65
	XICs	AT1G01300	APF2	Asp	E,PM	0.86	**0.64**	0.73	0.73
	XICs	AT4G34980	SLP2	Ser	E	0.81	**0.71**	0.87	1.16
	XICs	AT2G05920	SBT1.8	Ser	E,PM	0.82	**0.74**	0.71	0.77
Class 6	XICs	AT3G18490	ASPG1	Asp	E	0.93	0.81	0.85	**0.60**
	XICs	AT2G33530	SCPL46	Ser	E	1.22	0.97	0.89	**0.63**
	XICs	AT4G21650	SBT3.13	Ser	E	0.71	0.55 (–)	0.69	**0.65**
Class 7	XICs	AT5G08260	SCPL35	Ser	E	0.90	0.83	**0.66**	**0.65**
Class 8	XICs	AT1G09130	CLPR3	Ser	Pl	0.78	1.00	**0.41**	0.91

Plants were grown under high- (HN) or low- (LN) nitrate conditions. ^(1)^ Method for proteome analysis was XICs. ^(2)^ Protein names according to UniProt and TAIR. ^(3)^ Catalytic classes according to MEROPS: asp, aspartic proteases; met, metallo-proteases; ser, serine proteases. ^(4)^ Predicted subcellular localizations according to SUBA3 (SUBAcon) and Bio-Analytic Ressource for Plant Biology (Cell eFP Viewer). E, extracellular; PM, plasma membrane; Pl, plastid; V, vacuole. ^(5)^ Fold-change ratios were calculated by dividing protein abundance in *atg5* and in *atg5/sid2* by protein abundance in Col and in *sid2*, respectively. Entries in bold represent significantly decreased accumulations, all other entries are non-significant changes (*n* = 3; *P*<0.05; ANOVA and Tukey’s *post-hoc* test for multiple comparisons). (–) significant decrease in gene expression (Log_2_ fold-change of mRNA levels < –1.2 with *P*-value <10^–5^, rank product and FDR estimation).

**Fig. 2.**
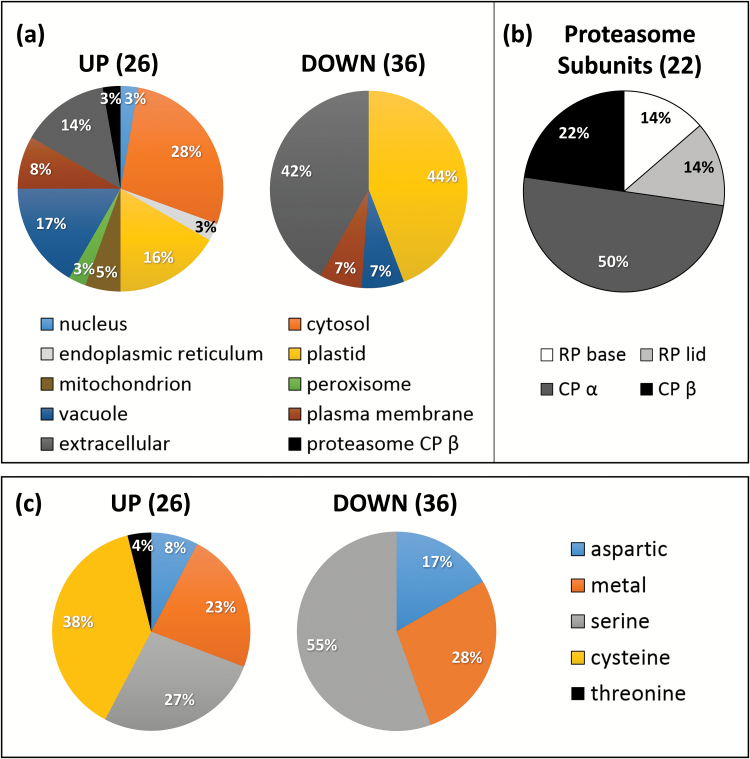
Predicted localization and catalytic classes of the proteases and proteasome sub-units in the autophagy-defective lines with significantly increased (up) or decreased (down) accumulation. Plants were grown under low- (LN) or high- (HN) nitrate conditions. Proteases and proteasome sub-units with significantly increased or decreased accumulation in *atg5* versus Col and/or *atg5/sid2* versus *sid2* under LN or HN, or both LN and HN are presented. The numbers of proteins considered for the percentage calculation are indicated in brackets. Significant differences were determined by ANOVA and Tukey’s *post-hoc* test for multiple comparisons (*P*<0.05, *n*=3). (a) Predicted subcellular localization of the increased and decreased proteases in the autophagy-defective lines using SUBAcon (SUBA3 database) and Cell eFP Viewer (Bio-Analytic Ressource for Plant Biology). Only catalytic proteasome sub-units (CPβ) are taken into account here. (b) A total of 22 20S core-protease (CP) and 19S regulatory-particle (RP) sub-units were increased in the autophagy-defective lines: The diagram represents their proportions. (c) Catalytic classes of the increased and decreased proteases in the autophagy-defective lines identified using the MEROPS database. Only the CP β sub-units of proteasome were considered here as active proteases.

The predicted localization at subcellular level of the proteases and protease-related proteins that either increased or decreased in accumulation in the autophagy-deficient lines are shown in Fig. 2a. Among the 47 protease-related proteins that increased in autophagy mutants, we found mainly proteasome subunits (22 proteins; Fig. 2b) and cytosol-localized proteases (28% of 26 proteases; Fig. 2a). Proteasome sub-units were both core proteases (α and β sub-units: 50% and 23%, respectively; Fig. 2b) and regulatory particles (14% for both base and lid complexes). Vacuole-, plastid-, and extracellular-predicted proteases were also identified (17%, 16%, and 14%, respectively; Fig. 2a). Accumulated proteases were mostly cysteine (38%), serine (27%), and metal proteases (23%) (Fig. 2c).

The 36 proteases that had decreased accumulation were mainly plastid-predicted (44%) and extracellular-predicted proteases (42%), and they belong to the serine (55%), metallo- (28%) and, to a lesser extent, aspartate (17%) proteases. There was no cysteine proteases or proteasome sub-units among the decreased proteins.

**Fig. 3.**
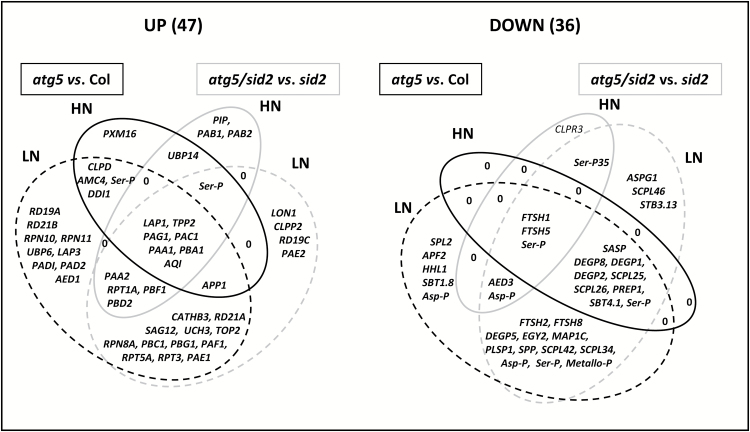
Venn diagrams of the proteases and proteasome sub-units with significantly increased (up) and decreased (down) accumulation in the autophagy-defective lines. Comparisons of *atg5* versus Col (black lines) and *atg5/sid2* versus *sid2* (grey lines) were performed on plants grown under low- (LN) or high- (HN) nitrate conditions. The number of proteins is indicated in brackets. Significant differences were determined by ANOVA and Tukey’s *post-hoc* test for multiple comparisons (*P*<0.05, *n*=3).

Venn diagrams of the proteases and proteasome sub-units that were significantly differentially accumulated in autophagy-deficient lines are presented in Fig. 3. Among the proteases that were accumulated compared to the controls (Table 1; Fig. 3a), seven were more abundant in both *atg5* and *atg5/sid2*, and under both nitrate conditions (Class1-up), four proteases were more abundant in *atg5/sid2* under both LN and HN, and also in *atg5* under LN (Class2-up), and 12 were specifically over-accumulated under LN in both *atg5* and *atg5/sid2*, i.e. in a SA-independent manner (Class3-up). The majority of the Class1-up, 2-up, and 3-up proteases were part of the proteasome machinery (Table 1), either threonine proteases or regulatory sub-units of the proteasome. Several proteasome-associated proteases such as LAP1, TOP2, and TPP2, or protein deubiquitinases such as UCH3 were also identified (Polge *et al.*, 2009). In Class1-up, only AQI (AQUAPORIN INTERACTOR) was not related to the proteasome, and in Class3-up, three well-known cysteine proteases CATHB3 (CATHEPSIN B3), RD21A (RESPONSIVE TO DEHYDRATION 21A), and SAG12 (SENESCENCE ASSOCIATED GENE 12) were found. Class4-up included proteases significantly accumulated compared to the controls in *atg5* only (i.e. SA-dependent) and under both LN and HN. It included the chloroplast CLPD subunit (also referred to as SAG15 or ERD1), the Arabidopsis METACASPASE 4 (AMC4), the ubiquitin-family aspartate protease DDI1 (DNA-DAMAGE INDUCIBLE 1), which is induced under cadmium stress, and an unknown serine protease (At1g50380). The proteins of Class5-up were specifically over-accumulated in *atg5* under LN. Class5-up included two proteasome non-catalytic sub-units (PAD1 and PAD2), two proteasome regulatory proteins (RPN10 and RPN11), the deubiquitinase UBP6, the leucine aminopeptidase LAP3, the two drought resistance-related cysteine proteases RD19A and RD21B, and the AED1 (APOPLASTIC, EDS1-DEPENDENT 1) aspartate protease. Class6-up, which represents proteases over-accumulated in *atg5/sid2* under high N, included PIP (PROLINE IMINOPEPTIDASE) and the two proteasome subunits PAB1 and PAB2. Class7-up grouped together four proteases over-accumulated in *atg5/sid2* under LN. Two of them are essential for mitochondria maintenance (LON1 and CLPP2), whilst the others are a proteasome subunit (PAE2) and a cysteine protease RD19C (RESPONSIVE TO DEHYDRATION 19C). Classes 8-up, 9-up, 10-up, and 11-up included UBP14 (UBIQUITIN-SPECIFIC PROTEASE 14; accumulated in *atg5* and *atg5/sid2* under HN only), PXM16 (metallo-protease of the M16 family; only accumulated in *atg5* under HN), APP1 (accumulated in all conditions and *atg* genetic backgrounds except in *atg5/sid2* under HN), and the unknown serine protease (At5g36210; accumulated compared to the controls in *atg5* and *atg5/sid2* under HN, and in *atg5/sid2* under LN). The ALEURAIN-LIKE PROTEASE, AALP (AT5G60360), detected by the shotgun LC-MS/MS proteome analysis was found to be slightly, but not significantly, increased in the *atg* lines under LN (see Supplemenatry Fig. S3).

The proteases that had decreased accumulation were also classified. The two FTSH1 and FTSH5 (FILAMENTATION TEMPERATURE SENSITIVE H 1 and 5) chloroplast proteases and an unknown vacuole serine protease (At4g36195) were significantly less abundant in both *atg5* and *atg5/sid2*, and under both LN and HN (Class1-down; Table 2). Class2-down contained two extracellular-predicted proteases [AED3 (APOPLASTIC, EDS1-DEPENDENT 3) and the product of *AT3G54400*]. Class3-down (down in *atg5* under both LN and HN, and in *atg5/sid2* under LN) principally included several serine proteases, almost all predicted in the extracellular space (such as SASP, SENESCENCE-ASSOCIATED SUBTILISIN PROTEASE), or in the plastids (such as the DEGRADATION OF PERIPLASMIC PROTEINS DEGP1, DEGP2, and DEGP8 proteases, and the PREP1 zinc metallo-protease). Class4-down clustered together proteases that were decreased in *atg5* and *atg5/sid2* only under LN. They were almost all plastid- (FTSH2, FTSH8, DEGP5) or extracellular-predicted, and they mostly belong to the serine and metallo-protease families. Class5-down grouped together proteases repressed in *atg5* under LN, and mainly included extracellular proteases, but also contained the HHL1 (HYPERSENSITIVE TO HIGH LIGHT 1) metallo-protease that is involved in the protection of photosystem II (Jin *et al.*, 2014). Class6-down contained proteases differentially repressed in the *atg5/sid2* mutant under LN. They are almost all extracellular-predicted and serine proteases except for ASPG1 (ASPARTIC PROTEASE IN GUARD CELL 1), which is extracellular-predicted, but also localizes in the endoplasmic reticulum in the guard cells (Yao *et al.*, 2012). Class7-down and Class8-down both contained only one member: the extracellular SCPL35 (decreased accumulation in *atg5/sid2* under LN and HN) and the chloroplast CLPR3 (decreased accumulation in *atg5/sid2* under HN), respectively.

From these results, it clearly appears that the protease-related proteins accumulated in *atg5* and/or *atg5/sid2* mostly belonged to the proteasome machinery and to the cytosolic or vacuole cysteine protease families. The proteases with decreased accumulation were mainly ones with chloroplast- or extracellular-predicted localizations and belonged mainly to the serine- and metallo-protease families. It should be noted that the decrease of chloroplast-predicted proteases in *atg5* was independent of SA (observed in both the Col and *sid2* backgrounds) and independent of N (observed under HN as well as under LN). It is thus likely that the decrease in these chloroplast-predicted proteases was not correlated to the slight senescence phenotype observed in *atg5* under low-nitrate conditions (Fig. 1b), but constitutes a more robust phenotype that was shared by the two autophagy-defective lines studied, independently of the nitrate conditions.

In order to determine whether changes in protease contents were linked to a modification of the transcription of their genes or to modifications in their protein turnovers, gene expression levels were evaluated using the transcriptomic data obtained by Masclaux-Daubresse *et al.* (2014), (Supplementary Dataset S1). Only the *CLPD*, *RD21B*, *AED1*, and *SAG12* genes were up-regulated in *atg5* and/or *atg5/sid2* (Table 1), and only *SBT3.13* was repressed in *atg5* under low nitrate (Table 2). Thus, the changes in the protease protein contents reported in Tables 1 and 2 were more likely due to modifications in their turnover rates.

### Proteasome and papain-like cysteine protease activities are higher in the *atg5* and *atg5/sid2* lines, but not serine protease activity

It is well known that protease activities can be regulated at several post-transcriptional levels. In addition, it has been shown in the case of the proteasome, for example, that autophagy could play a role in proteaphagy (Marshall *et al.*, 2015). Thus, in order to determine whether the accumulation or depletion of proteases detected in the *atg5* mutant lines caused modifications in protease activities, we explored changes in specific protease activities.

The degradation of the Rubisco large sub-unit (RBCL) at acidic pH was monitored using specific protease inhibitors according to the procedure described by Poret *et al.* (2016). We observed that RBCL degradation was higher in the two *atg5* lines relative to controls (see Supplementary Fig. S4a) and that degradation was inhibited when the E-64 cysteine-protease inhibitor or the aprotinine serine protease inhibitor were added before incubation of the extract (Fig. S4b).

**Fig. 4.**
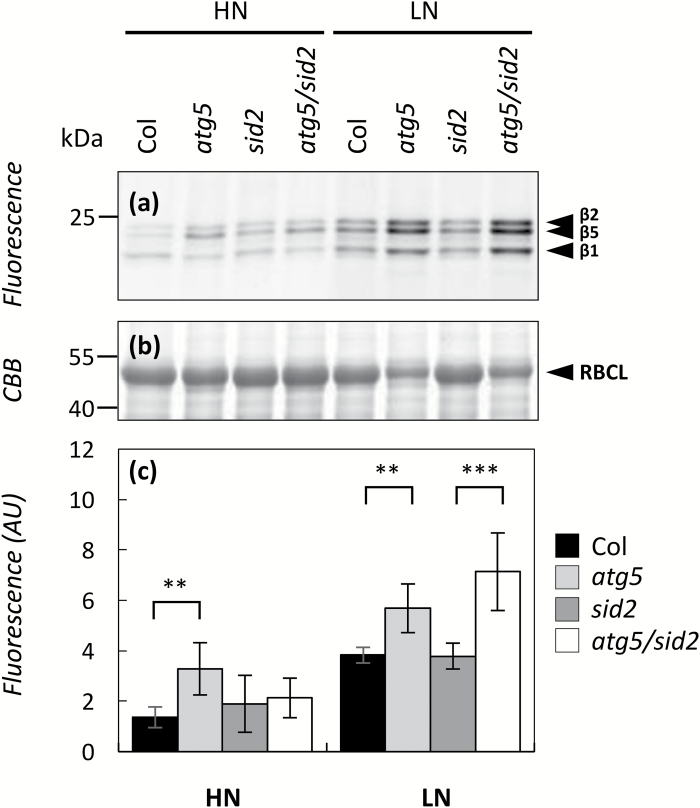
Proteasome activity is higher in autophagy-defective lines. Autophagy-defective lines (*atg5* and *atg5/sid2*) and their respective controls (Col and *sid2*) were cultivated under high- (HN) or low-(LN) nitrate conditions for 60 d. Soluble proteins were extracted from rosette leaves using water and labelled with MVB072 at pH 7.5 to detect proteasome activity. Proteasome activity (a) was determined by detecting the MVB072-specific fluorescence after separating 10 µg of proteins by SDS-PAGE. The β1, β2, and β5 catalytic sub-units are indicated by arrows. (b) The same SDS-PAGE gel was stained with Coomassie Brilliant Blue (CBB) to check for equal protein input. The Rubisco large sub-unit (RBCL) is indicated by an arrow. The fluorescence intensity (c) representative of the proteasome activity was quantified using Image Quant. Proteasome activity was measured on six biological repeats from two independent cultures. Means ±SD are shown (*n*=6) and significant differences between the autophagy-defective lines and the controls (i.e. *atg5* versus Col, and *atg5/sid2* versus *sid2*) are indicated: **P<0.01, ***P<0.001 (ANOVA and Tukey’s *post-hoc* test for multiple comparisons). The gels presented in this figure are representative of six biological replicates.

ABPP was performed in order to detect changes in the contents of active proteasome sub-units, active cysteine proteases, and active serine proteases (Morimoto and van der Hoorn, 2016). For the proteasome, the fluorescent probe MVB072 was added to the protein samples in order to derivatize the active proteasome protease sub-units. Fluorescent derivatized sub-units were then detected after SDS-PAGE separation (Fig. 4). MVB072 competition assays were carried out to estimate the probe’s specificity toward the proteasome sub-units (see Supplementary Fig. S5). Significant increases in the proteasome-active β1, β2, and β5 sub-units (encoded by *PBA1*, *PBB1/PBB2* and *PBE1/PBE2* genes respectively) were detected in *atg5* under HN, and in both *atg5* and *atg5/sid2* under LN. By contrast with PBA1 (proteasome-active β1subunit), no PBB1 or PBE2 subunits were identified in the shotgun analyses. These increases suggested that the proteasome activity was increased in autophagy-defective lines and especially under LN.

**Fig. 5.**
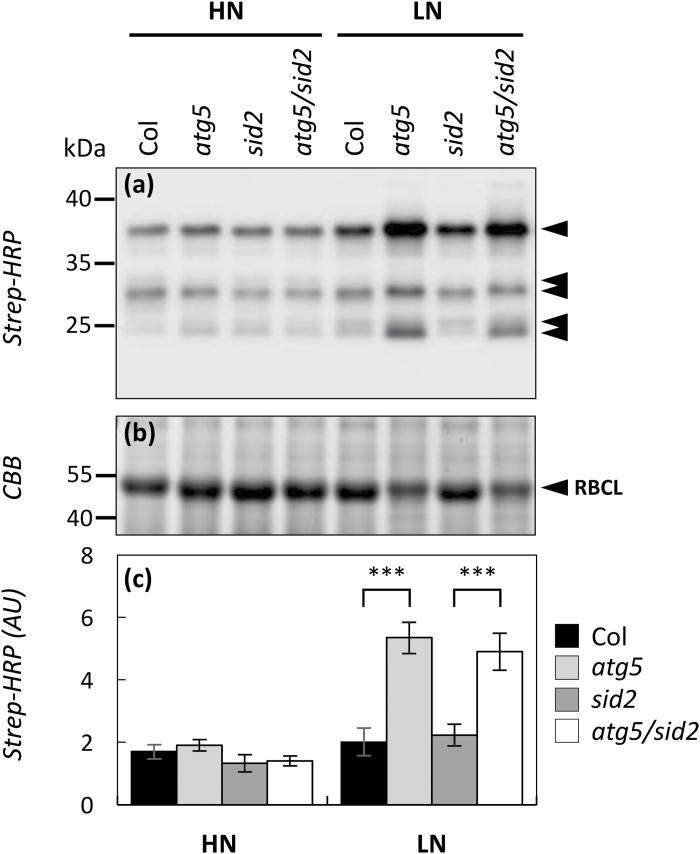
Activities of papain-like cysteine proteases (PLCPs) are higher in autophagy-defective lines grown under low-nitrate conditions. Autophagy-defective lines (*atg5* and *atg5/sid2*) and their respective controls (Col and *sid2*) were cultivated under high- (HN) or low- (LN) nitrate conditions for 60 d. (a) Active PLCPs labelled with DCG-04 at pH 5.5 (arrows) were detected using streptavidin-HRP chemiluminescence. (b) The same total amount of input proteins was loaded on each lane after incubation, as shown by the Coomassie Brilliant Blue (CBB)-stained gel. The Rubisco large sub-unit (RBCL) is indicated by an arrow. (c) The chemiluminescence signal intensity representative of the total PLCP activities was quantified using Image Quant. Activities of PLCPs were measured on three biological repeats. Means and ±SD are shown (*n*=3) and significant differences between the autophagy-defective lines and the controls (i.e. *atg5* versus Col, and *sid2atg5* versus *sid2*) are shown: ***P<0.001 (ANOVA and Tukey’s *post-hoc* test for multiple comparisons). The gels presented in this figure are representative of three biological replicates. Determination of PLCP activities was repeated on samples from two independent cultures, showing similar results.

In order to detect active papain-like cysteine proteases (PLCPs), DCG-04 was added to the protein samples before SDS-PAGE separation. The biotin-tag that linked to the active PLCP was detected on membranes by western blot analysis using streptavidin-HRP chemiluminescence (Fig. 5). Five bands were detected on the membranes and their chemiluminescence signal was clearly higher in *atg5* and *atg5/sid2* relative to Col and *sid2*, respectively, and especially under LN (Fig. 5a). The controls performed to demonstrate the specificity of the synthetized DCG-04 toward cysteine proteases are presented in Supplementary Fig. S6.

The active serine proteases were detected using desthiobiotin-FP, and at both acidic and neutral pH as serine proteases are localized in both vacuoles and chloroplasts (see Supplementary Figs. S7 and S8). Surprisingly, no differences between the *atg5* lines and control lines were detected. This suggests that the global serine protease activity was not different in the autophagy-defective lines compared to the control lines (Supplementary Fig. S8). Inconsistent with the data obtained using the aprotinine serine protease inhibitor on RBCL degradation at pH 5.5, this result suggests that the serine protease activities monitored using desthiobiotin-FP were not the same as those that degraded RBCL in our previous assay.

### Identification of active papain-like cysteine proteases in autophagy mutants.

**Fig. 6.**
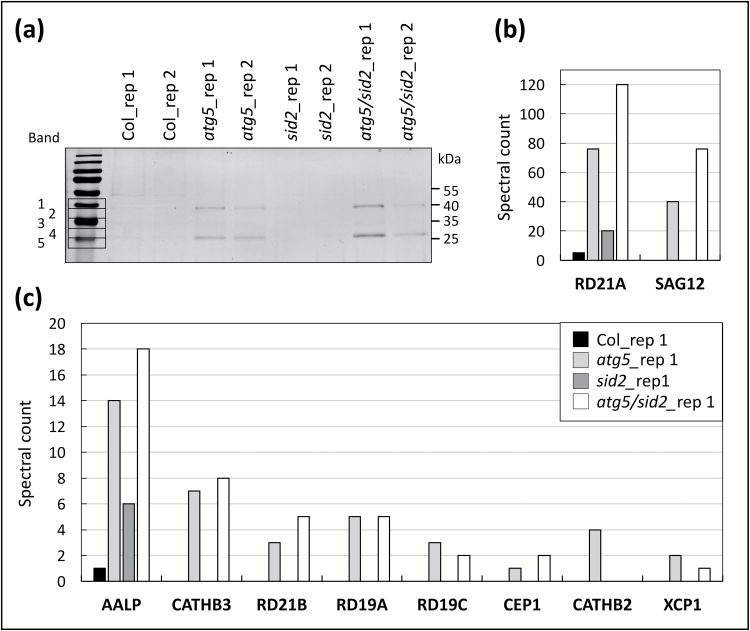
Identification of the active papain-like cysteine proteases (PLCPs) accumulated in autophagy-defective lines. Autophagy defective lines (*atg5* and *atg5/sid2*) and their respective controls (Col and *sid2*) were cultivated under low- (LN) nitrate conditions for 60 d. Soluble proteins from two biological replicates were extracted and labelled with DCG-04 at pH 5.5. Purified DCG-04-labelled proteins after pull-down for each genotype were separated on 12% SDS-PAGE gels and stained with silver nitrate (a); two replicates, rep1 and rep2, are shown. Proteins of molecular size between 40 and 25 kDa were excised from the gel (five bands) and analysed by LC-MS/MS. Ten active PLCPs were identified in all the genotypes and the two biological replicates. The total spectral counts obtained for each PLCPs are presented in (b) and (c). Similar results were obtained with the two replicates. Spectral counts in all the different excised bands presented in (a) are given in Supplementary Table S1.

Given that LC-MS/MS analyses showed that cysteine protease proteins accumulated in the autophagy mutants, and that DCG-04 revealed several active PLCP bands on membranes, a pull-down of active PLCPs was carried out using DCG-04. Co-precipitated PLCPs were separated on SDS-PAGE gels and streptavidin-HRP reactive bands were analysed using shotgun LC-MS/MS. This assay was only carried out on extracts from plants grown under LN conditions, as no differences had been detected on gels between genotypes when grown under HN (Fig. 5). In this experiment, spectral counting was used to determine the relative amounts of active PLCPs in the different genetic backgrounds (see Supplementary Table S1). The results showed that all the over-accumulated PLCPs previously identified in the autophagy-defective lines were active. The largest differences detected between the *atg5* and control lines after DCG-04 pull-down were for RD21A and SAG12 (Fig. 6b). Active CATHB3, AALP, RD21B, RD19A, and RD19C were also more abundant in *atg5* and *atg5/sid2* (Fig. 6c) Additional cysteine proteases were found that had not been identified by the shotgun LC-MS/MS whole-proteome analysis. They were CEP1 (CYSTEINE ENDOPEPTIDASE 1; At5g50260), which is involved in tapetum cell death and pathogen defence (Zhang *et al.*, 2014), CATHB2 (CATHEPSIN-LIKE B2 CASPASE-LIKE; At1g02305), which is senescence-induced (Ge *et al.*, 2016), and XCP1 (XYLEM CYSTEINE PEPTIDASE 1; At4g35350), which is a xylem cysteine peptidase (Avci *et al.*, 2008). It should be noted that *CEP1* was over-expressed at the transcriptional level in *atg5* and *atg5/sid2*, and under both HN and LN.

### Western blot identification of mature and immature forms of papain-like cysteine proteases

**Fig. 7.**
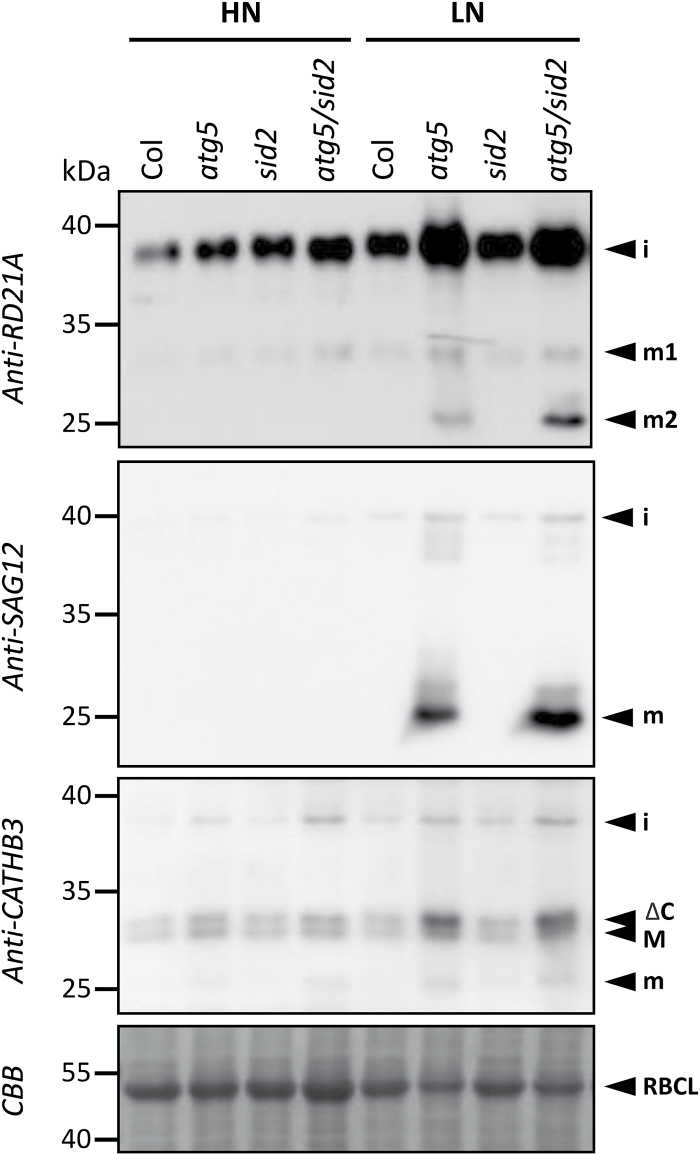
Identification of the mature and immature forms of papain-like cysteine proteases (PLCPs) using western blots. Autophagy-defective lines (*atg5* and *atg5/sid2*) and their respective controls (Col and *sid2*) were cultivated under high- (HN) or low- (LN) nitrate conditions for 60 d. Total proteins were extracted from rosette leaves using denaturing buffer. Proteins (5 µg) were separated on 12% SDS-PAGE gels and detected on protein blots using RD21A-, CATHB3-, and SAG12-specific antibodies. Arrows indicate the different PLCP forms: intermediate (i) and mature (m, m1, m2) for SAG12 and RD21A; immature pro-enzyme (i), intermediate form with C-terminal domain processed (ΔC), mature form (M), and short mature form (m) of CATHB3. Equal amounts of proteins were loaded in each lane, as shown by the Coomassie Brilliant Blue (CBB)-stained blot, on which the Rubisco large sub-unit (RBCL) is indicated by an arrow.

Antibodies raised against RD21A, SAG12, and CATHB3 were used in order to verify the accumulation of their processed forms and to monitor their immature forms in the atg5 lines under LN and also under HN (Fig. 7). The RD21A intermediate form (i) was detected in both *atg5* and control lines, and under both LN and HN. Its level was very high in all the lines, although it was higher in the *atg5* lines than in controls. The mature forms (m1 and m2; Fig. 7) were much less abundant than the intermediate forms, but still more abundant in the *atg5* lines than in controls. The m2 form was notably higher in the *atg5* lines under LN than under HN, and in *atg5/sid2* than in *atg5* under LN. For SAG12, the immature signal was weak, but higher in the *atg5* lines grown under LN. The mature form (m) was only observed in the *atg5* lines under low N and was much stronger than the immature form (i), in contrast with RD21A. All the CATHB3 mature and immature forms were more abundant in the *atg5* lines relative to the control lines, Col and *sid2*. The intermediate form with a processed C-terminal domain (ΔC) and the mature form (M) of CATHB3 were especially higher in the *atg5* lines than in the controls under low-nitrate conditions. Therefore, western blots confirmed that the active forms of RD21A, SAG12, and CATHB3 were more abundant in the autophagy-defective lines.

### Inhibitor proteins of cysteine proteases are differentially accumulated in autophagy-defective lines

Several protease inhibitors are small proteins that can form stable complexes with target proteases and block or prevent access to the active site. Among the long list of protease inhibitors available in the MEROPS database, three were found to be differentially accumulated in autophagy-defective lines compared to the controls (Table 3). PDI5 and Serpin-1, that can inhibit RD21A, had increased accumulation in *atg5* under both LN and HN conditions. PDI5 also accumulated in *atg5/sid2*. The extracellular CYS4 cysteine protease inhibitor was less abundant in *atg5/sid2* under LN.

**Table 3.** List of protease inhibitors differentially accumulated in *atg5* versus Col, and *atg5/sid2* versus *sid2*

Accession (TAIR10)	Name ^ (1) ^	Description ^ (2) ^	Predicted subcellular localization ^ (3) ^	Fold-change ^ (4) ^			
				*atg5*		*atg5/sid2*	
				HN	**LN**	**HN**	**LN**
AT1G21750	PDIL1-1/PDI5	protein disulfide isomerase	ER	**1.77**	**2.45**	**1.39**	**1.95**
AT1G47710	SERPIN-1	ser-protease inhibitor	C	**1.23**	**1.23**	1.11	1.13
AT4G16500	CYS4	cys-protease inhibitor	E	0.92	0.77	0.77	**0.55**

Plants were grown under high- (HN) or low- (LN) nitrate conditions. ^(1)^ Protein names according to UniProt and TAIR. ^(2)^ Description (TAIR10). ^(3)^ Predicted subcellular localizations according to SUBA3 (SUBAcon) and Bio-Analytic Ressource for Plant Biology (Cell eFP Viewer). C, cytosol; ER, endoplasmic reticulum; E, extracellular. ^(4)^ Fold-change ratios were calculated by dividing protein abundance in *atg5* and *atg5/sid2* by protein abundance in Col and *sid2*, respectively. Entries in bold cells represent significantly increased or decreased accumulations, all other entries are non-significant changes (*n* = 3; *P*<0.05; ANOVA and Tukey’s *post-hoc* test for multiple comparisons).

## Discussion

**Fig. 8.**
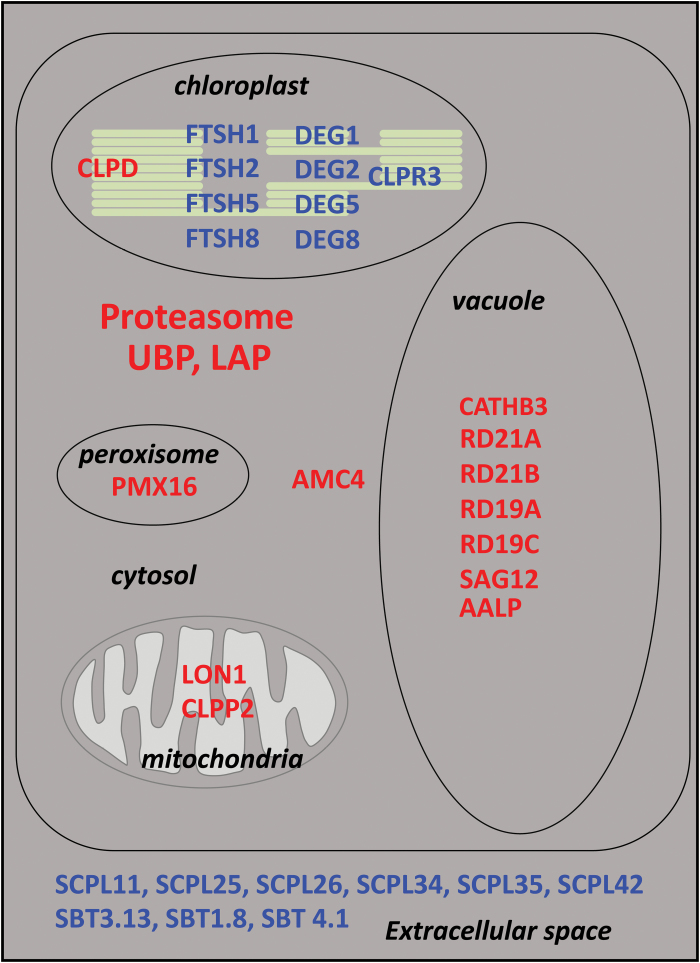
Schematic representation of the different proteases and protease-related proteins differentially accumulated in autophagy mutants and of their predicted cellular localization. The proteases with increased accumulation in *atg* are shown in red while those with decreased accumulation are shown in blue.

The aim of this study was to identify protease activities up-regulated in autophagy mutants in order to further determine their roles in nitrogen remobilization for seed filling (Guiboileau *et al.*, 2012). Shotgun LC-MS/MS and activity-based protein profiling (ABPP; Morimoto and van der Hoorn, 2016) analyses showed that, while protease genes were not transcriptionally induced or repressed in the autophagy mutants (*atg5* and *atg5/sid2*) relative to their controls (Col and *sid2*, respectively), many proteases were differentially abundant in the *atg* mutants (Fig. 8).

The proteases depleted in the *atg* mutants were mainly chloroplast- and extracellular-predicted proteases. Several chloroplast FTSH and DEG proteases were less abundant in the *atg5* lines (Kato and Sakamoto, 2010; van Wijk, 2015). FTSH-1, -2, -5, and -8 are the four major isomers of the chloroplast FTSH complexes involved in thylakoid maintenance (Yu *et al.*, 2004). Their decrease in the *atg* lines, especially under LN, suggested that thylakoid maintenance was affected. The DEGs ATP-independent serine-type proteases may be involved in the degradation of thylakoid lumen proteins and of PSII (Chassin *et al.*, 2002) and DEGP2 may be responsible for the endoproteolytic cleavage of the protein D1 (Haussühl *et al.*, 2001). Hence, together with the decrease of FTSH proteins, the decrease of DEGP-1, -2, -5, and -8 protein abundance suggests that thylakoid and photosystem maintenance are affected in *atg* lines. The number of chloroplast proteases with decreased accumulation in the autophagy-defective lines was slightly higher under nitrate starvation, but was not dependent on the leaf-senescence phenotype or on SA synthesis. The lower abundance of chloroplast proteases in the autophagy-defective lines suggests that chloroplasts could be less abundant or less active in the *atg5* lines than in the wild-type, thus explaining the lower carbon, starch, and sugar contents measured in *atg5*, *atg9*, and *atg18a*-RNAi by Guiboileau *et al.* (2013) and Masclaux-Daubresse *et al.* (2014).

In addition to chloroplast proteases, many extracellular-predicted subtilisin-like proteases (subtilases; SBTs) and extracellular-predicted serine carboxypeptidase-like proteases (SCPLs) were decreased in the autophagy-defective lines. The SBTs have mainly been described in plant–pathogen or pest-defence responses (Figueiredo *et al.*, 2014). The SCPLs are more likely involved in secondary metabolism and could also have a role in plant defence (Liu *et al.*, 2008; Mugford and Osbourn, 2010). The current understanding of the function of SBTs remains very limited, although a role for some of them in plant immune priming has been proposed (Figueiredo *et al.*, 2014). It is difficult to explain why the *atg5* and *atg5/sid2* lines had reduced amounts of extracellular SBTs and SCPLs. Dysfunction in exocytosis in the *atg* mutants could be one explanation, although a relationship between exocytosis and autophagy remains to be clearly demonstrated in plants (Wang *et al.*, 2010; Lin *et al.*, 2015; Münz, 2017). Nevertheless, the lower extracellular content of SBTs in *atg5* might explain the higher susceptibility of the autophagy mutants to necrotrophic pathogens, as reported by several studies (Lai *et al.*, 2011; Lenz *et al.*, 2011). Martinez *et al.* (2015) recently reported that the extracellular senescence-induced SASP subtilisin protease was involved in branching; the authors hypothesized that SASP could be involved in the processing of extracellular apoplastic signals repressing inflorescence branching and delaying leaf senescence. Although we found decreased accumulation of SASP in the *atg5* lines, no branching phenotype could be observed, but senescence was possibly enhanced.

Among the proteases with increased accumulation in the autophagy-defective lines, it was noticeable that many were proteasome sub-units and that several proteins were related to the ubiquitin proteasome system (UPS), such as LAP1, TOP2, TPP2, UCH3, and UBP14. The UPS and autophagy are two major pathways that degrade most of the cellular proteins of eukaryotic cells. It is considered that while the proteasome is responsible for the turnover of short-lived proteins, autophagy is more specifically dedicated to the degradation of long-lived or aggregated proteins (Lilienbaum, 2013, and references therein). It appears that the UPS and autophagy are tightly controlled and co-ordinated. Several studies have suggested that autophagy may act as a back-up system, thereby assisting degradation in case the UPS is overloaded (Nedelsky *et al.*, 2008). It seems that most of the mis-folded soluble proteins that are preferentially degraded by the UPS in cases of endoplasmic reticulum (ER) stress can be degraded through the autophagy pathway if the proteasome capacity is exceeded (Johnston *et al.*, 1998). Several studies have also suggested that the proteasome itself could be degraded by autophagy (Waite *et al.*, 2016). In plants, the accumulation of several proteasome sub-units in autophagy-defective mutants has been reported. Marshall *et al.*, (2015) showed that RPN10 could act as a selective autophagy receptor that targets ubiquitinated and inactive 26S proteasomes and binds to ATG8 for autophagy-mediated degradation. We also found that several proteasome sub-units were more abundant in *atg5*, especially under LN, including RPN10 (Table 1). This suggests that proteaphagy was increased under LN and was compromized in *atg5*. It thus seems that low-N conditions that trigger N remobilization (Masclaux-Daubresse and Chardon, 2011) can induce selective proteaphagy (Waite *et al.*, 2016; Zientara-Rytter and Sirko, 2016).

With regards to the proteasome, our ABPP assay showed that active β1, β2, and β5 catalytic subunits of the proteasome were more abundant in *atg5* under LN and HN and also in the *atg5/sid2* defective line under LN. This suggested that the activity of the proteasome was globally higher in the autophagy-defective lines. It has been shown that ER stress is increased in plant autophagy mutants (Munch *et al.*, 2014; Yang *et al.*, 2016). A role for the proteasome in the endoplasmic reticulum-associated degradation of mis-folded proteins under ER stress has been demonstrated in mammals (Lee *et al.*, 2001). We can therefore suspect that an increase in proteasome activity in atg5 and *atg5/sid2* could be linked to ER stress.

In addition to the accumulation of UPS proteins, increases of several cysteine, serine, and metallo-proteases were observed in the *atg5* and *atg5/sid2* lines. Activity measurements, performed using protease inhibitors, indicated that cysteine protease activities were significantly different between the *atg5* and *atg5/sid2* lines and the Col and *sid2* controls. As they are suspected to play a fundamental role in N remobilization during senescence and in response to nitrate shortage, we then focused on the papain-like cysteine proteases (PLCPs; Avice and Etienne, 2014; Diaz-Mendoza *et al.*, 2016; Pružinská *et al.*, 2017). Pull-down using DCG-04 showed that active PLCPs were more abundant in the *atg5* and *atg5/sid2* lines (Table 1). Active SAG12, RD21A, CATHB3, and AALP were strongly enriched in *atg5* and *atg5/sid2*, as well as RD19A, RD19C, CEP1, CATHB2, and XCP1, although to a lower extent. With the exception of *SAG12* and *CEP1*, PLCP genes were not differentially expressed in *atg5* versus Col or *atg5/sid2* versus *sid2*. This absence of a significant change at the transcriptional level suggests that the accumulation of PLCPs in *atg5* and/or *atg5/sid2* was mainly due to higher translation or post-translational maintenance. Most of the PLCPs identified were predicted in the vacuole, except for RD21s that have been also found in ER-bodies (Hayashi *et al.*, 2001), SAG12 that was detected in senescence-associated vacuoles (Otegui *et al.*, 2005), and CEP1 that seems only to be located in the ER (Zhang *et al.*, 2014). Many of the PLCPs, such as RD21s, RD19s, and CATHB3, transit through the ER before being delivered into the vacuole lumen where they play their protease functions. Whether autophagy could play a role in the trafficking of these proteases from the ER to the vacuole, as was suggested for seed-storage proteins (Michaeli *et al.*, 2014), is unknown. The western blots performed using the RD21A, CATHB3, and SAG12 antibodies revealed that both mature and immature forms were accumulated the *atg5* and *atg5/sid2* lines, thus suggesting that there was no defect in protease maturation or trafficking in the autophagy mutants.

All the PLCPs identified in this study are known proteases. They have been described in several reports but their regulation, maturation, and physiological roles remain largely enigmatic (van der Hoorn, 2013; Rogers, 2013; Iglesias-Fernández *et al.*, 2014; Zhang *et al.*, 2014; Pathirana *et al.*, 2017). Several are known to be transcriptionally induced during leaf senescence, such as *SAG12*, *CATHB3*, *CATHB2*, *AALP*, *CEP1*, *RD21A*, *RD21B*, *RD19A*, and *RD19C* (Supplementary Table S2; Noh and Amasino, 1999; Havé *et al.*, 2017; Pružinská *et al.*, 2017). However, as none of them except *SAG12* and *CEP1* were transcriptionally induced in the autophagy-defective lines relative to the controls, the possibility that these PLCPs were more abundant in *atg5* and *atg5/sid2* due to early senescence is weak. All these PLCPs were found to be more abundant in both *atg5* and *atg5/sid2* under LN but not under HN, and hence it is highly possible that they are involved in N r emobilisation processes (Masclaux-Daubresse and Chardon, 2011), and are induced under low N and especially when autophagy is impaired. These proteases are thus good candidates as enzymes involved in the N-recycling pathways that lead to the remobilization of organic nitrogen forms from the source leaves to the sink leaves and to the seeds (Desclos *et al.*, 2009). Their potential roles as back-up proteolytic systems to compensate for weaknesses in autophagy, or as the last step of an autophagy process and cargo degradation inside the vacuole, need verification. Pružinská *et al.* (2017) have recently shown that only knockout lines lacking AALP present delayed leaf senescence under dark stress, in contrast with the *rd21a*, *rd21b*, *sag12*, and *cathb3* mutants. It therefore seems that AALP may be the best candidate for playing a role in N remobilization, together with, or in parallel to, autophagy.

It is well known that autophagy mutants exhibit spontaneous regions of cell death on their leaves with ageing (Masclaux-Daubresse *et al.*, 2014). The role of the PLCPs identified here in this phenotype of the autophagy mutants can be questioned (Lampl *et al.*, 2013; Iglesias-Fernández *et al.*, 2014; Cai *et al.*, 2018; Zhang *et al.*, 2014). Indeed, several reports suggest that AMC4, CATHB3, PBA1, and RD21s might play a role in the control of cell death (Watanabe and Lam, 2011; Cai *et al.*, 2018), either as cell death-promoting or longevity factors. Interestingly, we found that AMC4 accumulated in *atg5* but not in *atg5/sid2* whatever the nitrate conditions, while RD21A, CATHB3, and SAG12 accumulated in both *atg5* and *atg5/sid2* but only under LN. These discrepancies in accumulation patterns possibly distinguish the different functions of these proteases. Proteases promoting death should be independent of nitrate but SA-dependent, such as AMC4, while the N-remobilization proteases should be SA-independent but nitrate-dependent, such as RD21A, CATHB3, and SAG12. Hypotheses such as these need further investigations with mutants in order to be confirmed.

Changes in protein inhibitors of proteases were also identified. Increases in PDIL1/PDI5 and in Serpin-1 proteins suggested additional controls exerted on the RD21 proteases in the *atg5* and *atg5/sid2* lines to moderate their activity (Lampl *et al.*, 2013; Rustgi *et al.*, 2017). The decrease of CYS4 suggests that its unknown cysteine protease partners are repressed in the autophagy-defective lines (Je *et al.*, 2014).

In conclusion, we identified several proteases that are differentially accumulated in autophagy-defective lines compared to their controls, and that could explain the phenotype of autophagy mutants such as the spontaneous regions of cell death on the leaves and the lower carbon and sugar contents due to reduced chloroplast maintenance. Many of the PLCPs identified could play a compensatory role for protein degradation and nutrient recycling, especially under low nitrate nutrition. Further investigations and biochemical studies are required using protease and autophagy mutants to clarify their contributions.

## Supplementary data

Supplementary data are available at *JXB* online.

Methods S1–S3. Phenotyping display and growth conditions; details of the shotgun proteomics analysis; and details of the shotgun LC-MS/MS analysis of pulled-down PLCPs.

Fig. S1. α-mRD21A and cathepsin B3-N Term-1610N antibodies are specific for Arabidopsis RD21A and cathepsin B3, respectively.

Fig. S2. Salicylic acid concentrations in *atg5-1/sid2* and *atg*5-2/NahG grown under low-nitrate conditions.

Fig. S3. ALEU protease amounts tend to increase in autophagy-defective mutants.

Fig. S4.: Rubisco degradation in *atg* defective lines and controls.

Fig. S5. MVB072 competition assay to estimate the probe’s specificity toward proteasome sub-units.

Fig. S6. DCG-04 competition assay to estimate the probe’s specificity toward PLCPs.

Fig. S7. Desthiobiotin-FP competition assay to estimate the probe’s specificity toward serine proteases.

Fig. S8. Serine protease activities are stable in autophagy mutants.

Table S1. Details of spectral counts for PLCP peptides in Col, *sid2*, *atg5*, and *atg5/sid2*.

Table S2.: Senescence up- and down-effects on the transcription of protease and protease-inhibitor genes.

Dataset S1. Transcriptomic data obtained by Masclaux-Daubresse *et al.* (2014).

Supplementary Dataset

Supplementary Material

Supplementary Methods

## Author contributions

MH performed all the experiments with technical help from ED. GC, FS, and TB ran the LC-MS/MS analyses. MH and TB collected and analysed the LC-MS/MS data. BC-B synthetized the DCG-04 probe. PG provided the CATHB3 antibody and performed controls on *cathb3* mutants to certify antibody specificities. ND and PR provided the anti-RD21A antibody and performed controls on the *rd21a* mutant and recombinant mRD21A protein. MZ and LR participated in the design of the biochemical experiments and in discussions. AL and J-CA designed and performed the Rubisco degradation assays. CM-D designed and supervised the research. MH and CM-D interpreted the results and wrote the manuscript.
