# Supplementary material for: Increases in activity of proteasome and papain-like cysteine protease in Arabidopsis autophagy mutants: back-up compensatory effect or cell-death promoting effect?
Source: J Exp Bot. 2018 Jan 27;69(6):1369–85. doi: 10.1093/jxb/erx482 (PMC6037082; doi:10.1093/jxb/erx482)
Supplement: Supplementary Material [file erx482_suppl_supplementary_material.pdf]

Increase of proteasome and papain-like cysteine protease activities in autophagy mutants: backup compensatory effect or pro cell-death effect?

Marien Havé[1], Thierry Balliau[2], Betty Cottyn-Boitte[1], Emeline Dérond[1], Gwendal Cueff[1], Fabienne Soulay[1], Aurélia Lornac[3], Pavel Reichman[4], Nico Dissmeyer[4], Jean-Christophe Avice[3], Patrick Gallois[5], Loïc Rajjou[1], Michel Zivy[2], Céline Masclaux-Daubresse[1*]

Supporting informations

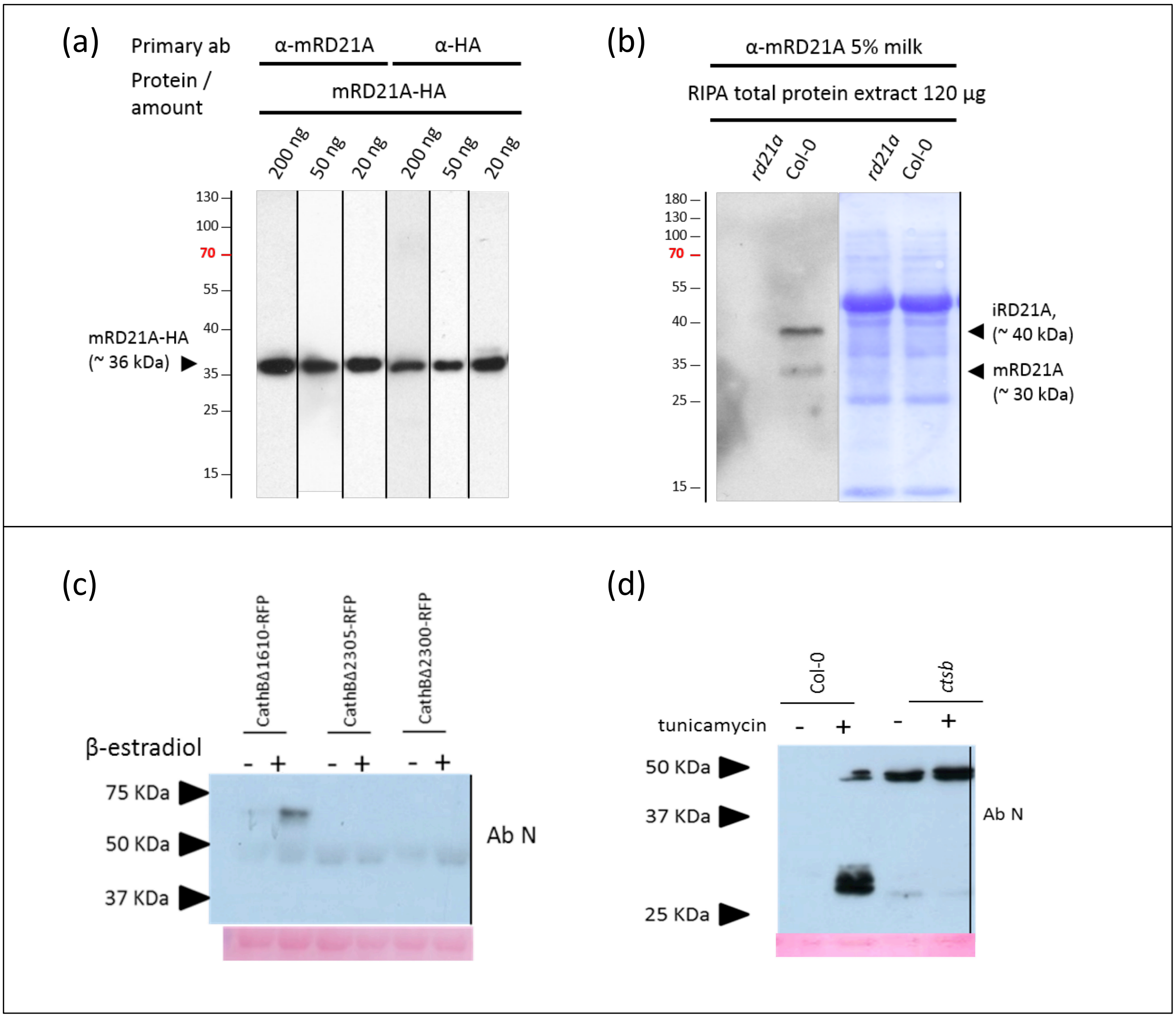

Fig. S1: α-mRD21A and cathepsin B3-N Term-1610N antibodies are specific for *Arabidopsis* RD21A and cathepsin B3, respectively. (a) RD21A recombinant protein was purified from *E. coli* using a fusion with the maltose binding protein. The mRD21A version was created via *in-vitro* cleavage by TEV protease. Cleavage by-products and the TEV protease itself were then removed through metal chelate affinity chromatography via His-tag. Cleaved purified proteins were separated on 10% SDS-PAGE and transferred to PVDF membrane using semi-dry blotting. Membranes were blocked for 1.5 h at room temperature or overnight at 4°C. Membranes were then incubated overnight at 4°C with primary antibodies (1:1000 diluted in 5% milk in TBS-T): the α-mRD21A (sheep) polyclonal IgG (0.4 mg/ml), raised against peptide RDELPESIDWRKKGC and the HA-probe polyclonal IgG Y-11 antibody (rabbit, Santa Cruz Biotechnology, catalog #: sc-805). Membranes were washed and incubated with the seconday antibody for 1 h at room temperature. Secondary antibodies used were : the anti-sheep (rabbit) IgG (H+L) HRP conjugate (Thermo Scientific, catalog #: 31480, used dilution: 1:10 000 in 5% milk in TBS-T) for RD21A and anti-rabbit (goat) IgG HRP conjugate (Santa Cruz Biotechnology, catalog #: sc-2004, lot# F2215, used dilution: 1:5 000 in 5% milk in TBS-T) for HA-probe. Anti-mRD21A and Anti-HA antibodies were probed in parallel. Vertical lines indicate splicing of several images of the ECL detection. All blots treated in parallel in the same experiment. **(b)** Total proteins were extracted from 7 day old *Arabidopsis* seedlings from *rd21a* (SALK_090550 mutant, Wang *et al*. 2008) and Col-0 wild type using RIPA buffer. Protein amount was measured using a Direct Detect Infrared Spectrometer (Millipore). Equal amount of proteins were loaded in each lane and were separated on 12% SDS-PAGE and blotted the same way as the recombinant proteins (a). After immunoblot analysis, membrane were stained with Coomassie Brilliant Blue for loading control. Note that milk blocking buffer is more suitable for specific RD21A detection than BSA blocking buffer that increase unspecific protein recognition. Milk blocking buffer was used in Fig. 7.

Fig. S1 legend continue

(c) The CATHB3 rabbit antibody was generated against a peptide matching the N-terminal sequence of the mature *Arabidopsis* cathepsin B3 (At4g01610). Using the cDNAs of *CATHB2* and *3* and the genomic clone of *CATHB1*, cathepsin B constructs were made where the C-term pro-domain was removed before fusing to RFP using a β-estradiol-inducible vector, creating CathBΔ1610-RFP for CATHB3; CathBΔ2305-RFP for CATHB2 and CathBΔ2300-RFP for CATHB1. Each construct was agro-infiltrated into tobacco leaves and co-infiltrated with (+) or without (-) β-estradiol. One day after induction, soluble proteins were extracted from infiltrated areas and analysed using western blot and the antibody CATHB 3-N Term-1610C (Ab N) at 1:1000. Ponceau red is used as a loading control. The cathepsin B3-N Term-1610N only detected the CathBΔ1610-RFP fusion (approx. 70kD). **(d)** One-week-old *Arabidopsis* seedlings, WT or triple cathepsinB mutant ≠62 (ctsb) were untreated (-) or treated (+) with 15μg/ml tunicamycin to induce AtCATHB3 accumulation. Soluble proteins were extracted after 3 days and subjected to western analysis using the antibody cathepsin B3-N Term-1610C (Ab N) at 1:1000. Ponceau red is used as a loading control. The reacting band at 50kD in panel (d) is interpreted as background for this antibody.

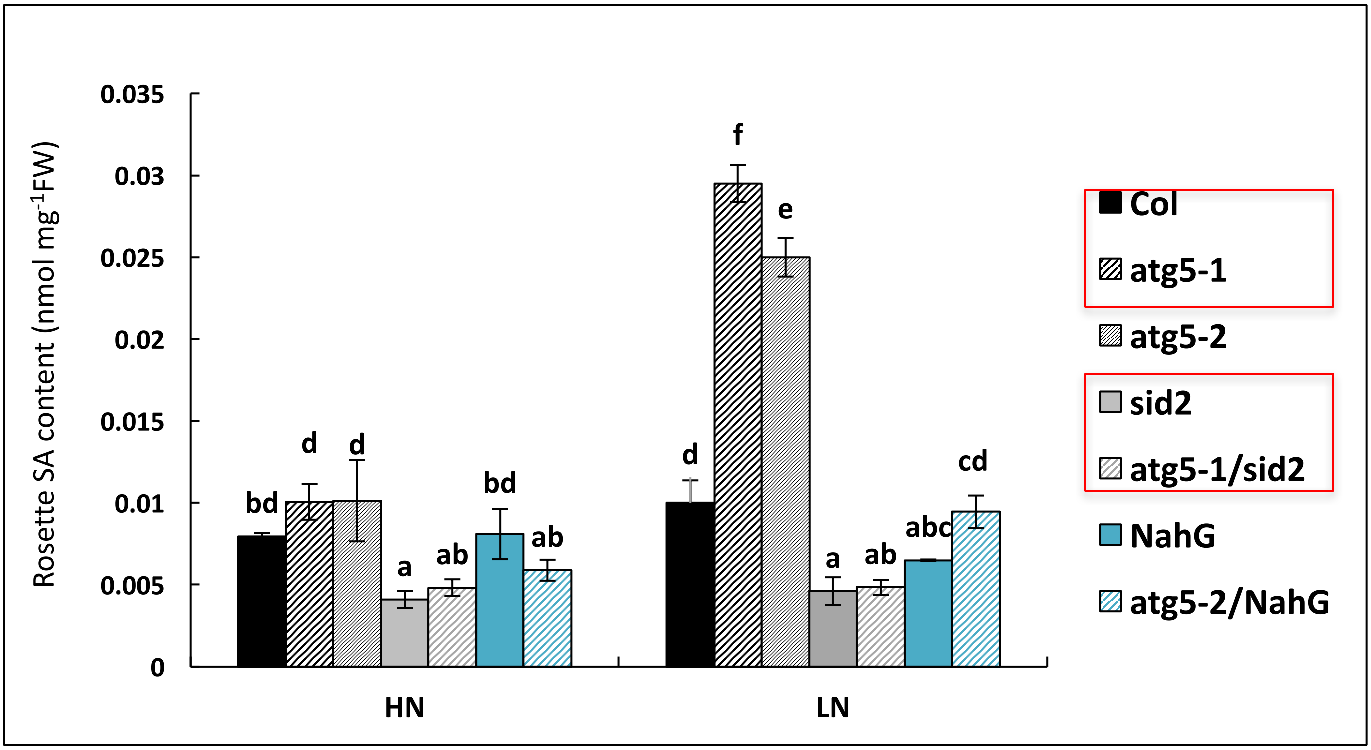

Fig. S2: Salicylic acid concentration in *atg5-1*/*sid2* and *atg*5-2/NahG grown under low nitrate conditions.

Autophagy defective lines (*atg5-1, atg5-2, atg5-1*/*sid2* and *atg5-2*/*NahG*) and their respective controls (Col, Col, *sid2* and *NahG*) were cultivated under high (HN) or low (LN) nitrate conditions for 60 days. Metabolome profiling of rosette leaves was analyzed as described in Masclaux-Daubresse *et al.*, (2014) using GC-MS. Salicylic acid was quantified according to standard. Histogram shows that the concentration of salicylic acid in the rosette leaves of *atg5-1*/*sid2* was as low as in the rosette of *sid2* under both HN and LN while salicylic acid concentration in *atg*5-2/NahG rosettes was significantly higher than in NahG under LN and also higher than in *sid2*.

Values are means±SD of 3 biological replicates. Letters indicate homogenous subsets as determined by ANOVA and Tukey's *post-hoc* test for multiple comparison, $P<0.05$. Red rectangles indicate the genotypes used in our study.

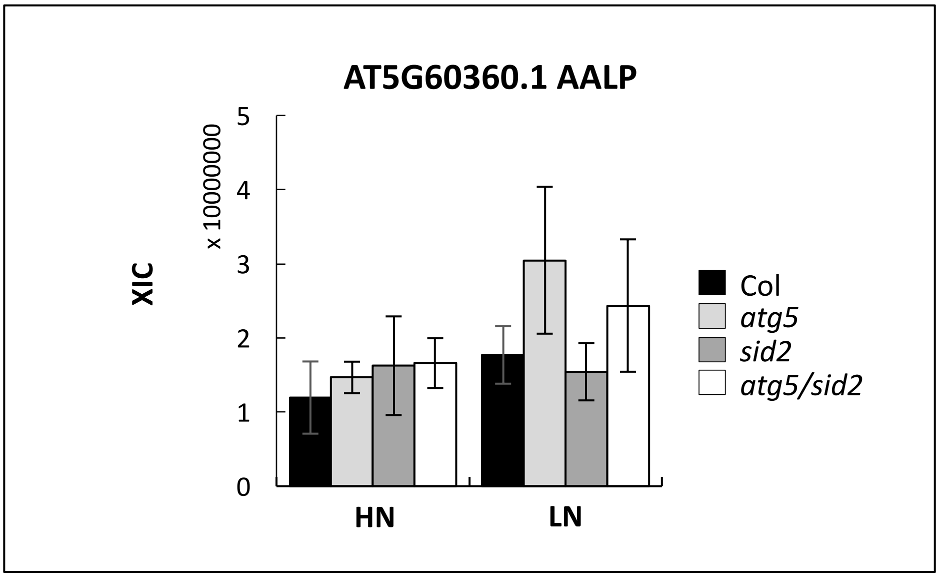

Fig. S3: ALEU protease amount tend to increase in autophagy defective mutants. Autophagy defective lines (*atg5* and *atg5*/*sid2*) and their respective controls (Col and *sid2*) were cultivated under high (HN) or low (LN) nitrate conditions for 60 days. Total proteins were extracted from rosette leaves using TCA/acetone and suspended in denaturing buffer. Proteins were then identified by LC-MS/MS and quantified using mass chromatograms using MassChroQ. Values are means± SD of 3 biological replicates. No significant differences from controls (i.e; *atg5 versus* Col *and atg5*/*sid2 versus sid2* were observed by ANOVA and Tukey's *post-hoc* test for multiple comparison.

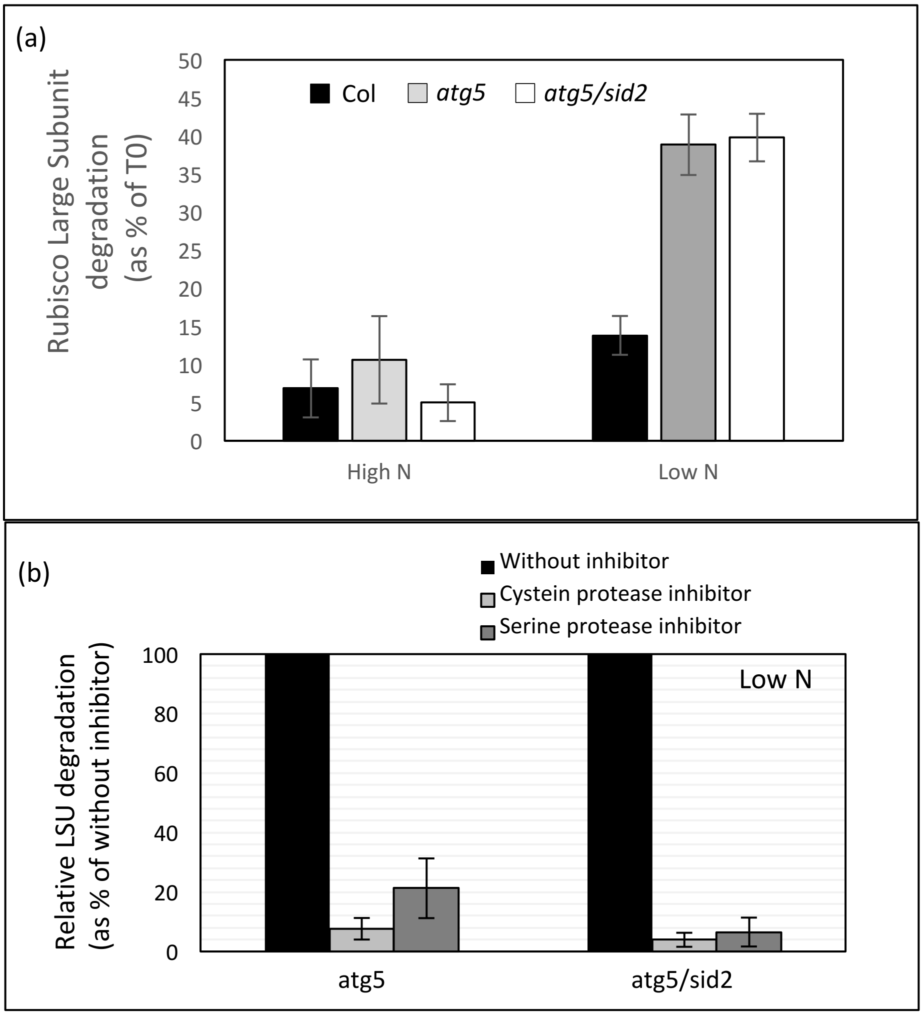

Fig. S4: RuBisCo degradation in *atg* defective lines and controls. (a) Degradation of the Rubisco large subunit in *atg* and control was measured as explained below, on plants grown under low or high nitrate conditions. Means and SD of 3 replicates are shown. *sid2* and Col controls showed similar degradation % and were grouped as control in the graph. **(b)** Effects of cysteine protease and serine protease inhibitors on Rubisco large subunit degradation in *atg5* and *atg5*/*sid2* plants grown under low nitrate conditions. Means and SD of 3 replicates are shown.

The degradation of RuBisCo large subunit (RBCL) by proteases within the soluble protein extract was studied with or without inhibitors of specific protease classes according to Girondé et al. (2015) [BMC Pant Biol, 21 15-59]]. RBCL was used as a target of proteolysis. Protease activities were determined at pH 5.5. Protein extracts were incubated in the presence or absence of E-64 or aprotinin. Incubation was performed for 30 min at 37 ◦ C under gentle agitation. No-inhibitor control was performed. To determine the initial quantity of RBCL (T0), the protein extract was treated without inhibitor and the proteolytic reaction was stopped immediately by adding 1 mL of ice-cold acetone. Soluble protein extracts were separated on a 4–15% gradient in SDS-PAGE precast Stain-free gels (Mini-PROTEAN ® TGX TM Stain Free, Bio-Rad, Marne- la-Coquette, France) and scanned under UV light with a Gel Doc TM EZ scanner (Bio-Rad, Marne-la-Coquette, France). The amount of RBCL (expressed as volume) was quantified by using ImageLab TM software (Bio-Rad, Marne-la-Coquette, France) according to the manufacturer's instructions. The percentage of RBCL degradation was calculated as the difference in quantity between non incubated and incubated samples without inhibitors. The percentage of inhibition due to the different inhibitors was calculated as the difference in degradation without or in the presence of inhibitors.

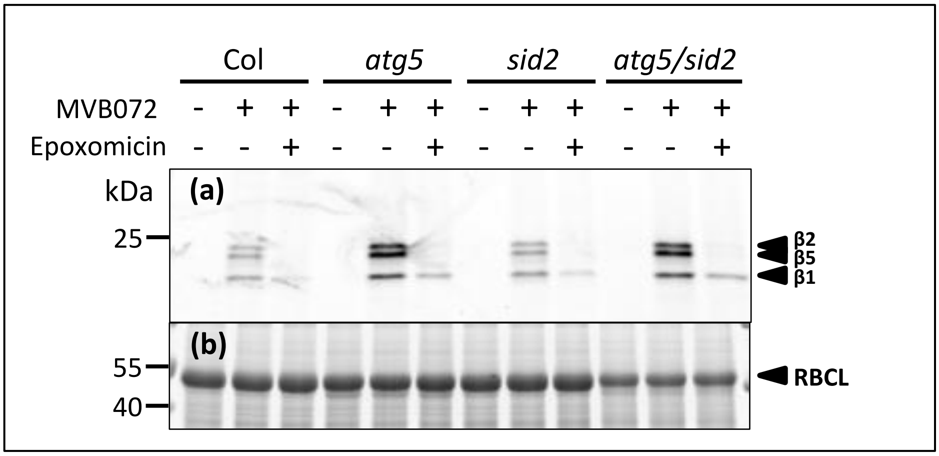

Fig. S5: MVB072 competition assay to estimate the probe's specificity toward proteasome subunits .

Autophagy defective lines (*atg5* and *atg5*/*sid2*) and their respective controls (Col and *sid2*) were cultivated under high (HN) or low (LN) nitrate conditions for 60 days. Soluble proteins were extracted from rosette leaves using water and protein extracts from HN and LN were pooled at equal protein amount for each genotype. To estimate MVB072 specificity toward proteasome subunits, samples were labelled with MVB072 or pre-treated with Epoxomicin then labelled with MVB072 (competition assay) or simply incubated with the probe's solvent (DMSO) as a no probe control (NPC). Proteasome activity (a) was revealed detecting the MVB072 specific fluorescence after separating 10 μg of proteins by SDS-PAGE. (b) The same SDS-PAGE gel (b) was stained with Coomassie brilliant blue (CBB) to check for equal protein input. Rubisco large subunit (RBCL) is indicated by arrow. Labelling with MVB072 allow the detection of three fluorescent bands corresponding to the b1, b2 and b5 catalytic subunits (indicated by arrows) as observed in Gu et al. 2010 and Poret et al. 2016. Those signals are absent in the NPC and heavily suppressed upon epoxomicin treatment showing good specificity of the MVB072 probe.

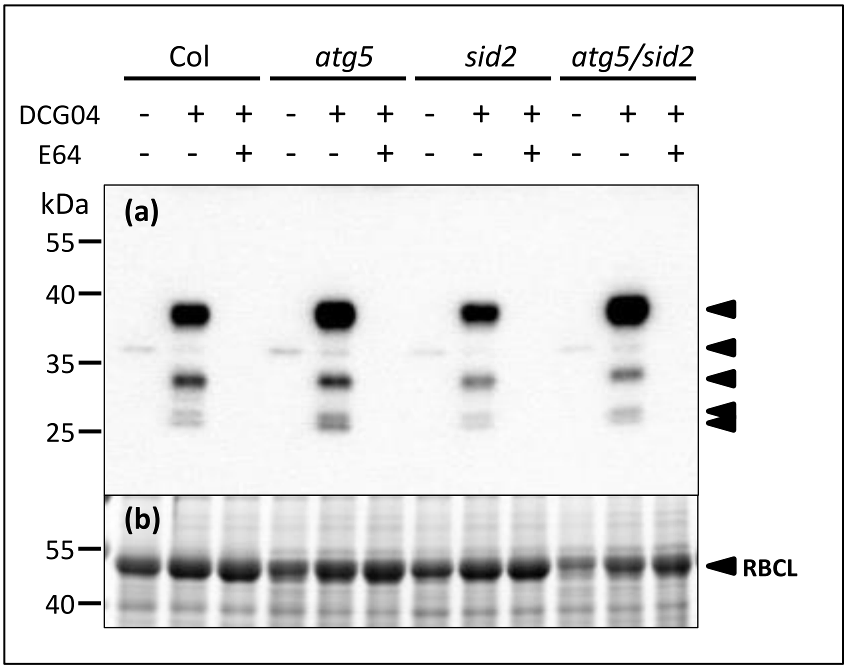

Fig. S6: DCG04 competition assay to estimate the probe's specificity toward PLCPs.
Autophagy defective lines (*atg5* and *atg5*/*sid2*) and their respective controls (Col and *sid2*) were cultivated under high (HN) or low (LN) nitrate conditions for 60 days. Soluble proteins were extracted from rosette leaves using water and protein extracts from HN and LN were pooled at equal protein amount for each genotype. To estimate DCG04 specificity toward PLCPs, samples were labelled at pH 5.5 with DCG04 or pre-treated with E64 then labelled with DCG04 (competition assay) or simply incubated with the probe's solvent (DMSO) as a no probe control (NPC). PLCPs activities (a) were detected using streptavidin-HRP chemiluminescence after separating 10 μg of proteins by SDS-PAGE. (b) The same SDS-PAGE gel was stained with Coomassie brilliant blue (CBB) to check for equal protein input. Rubisco large subunit (RBCL) is indicated by arrow. A band around 35 kDa is detected in the NPC suggesting the presence of an endogenously biotinylated protein. Labelling with DCG04 allow the detection of bands between 25 and 40 kDa as observed in van der Hoorn et al. 2004. These signals were completely absent in the competition assay demonstrating the specificity of DCG04 toward PLCPs.

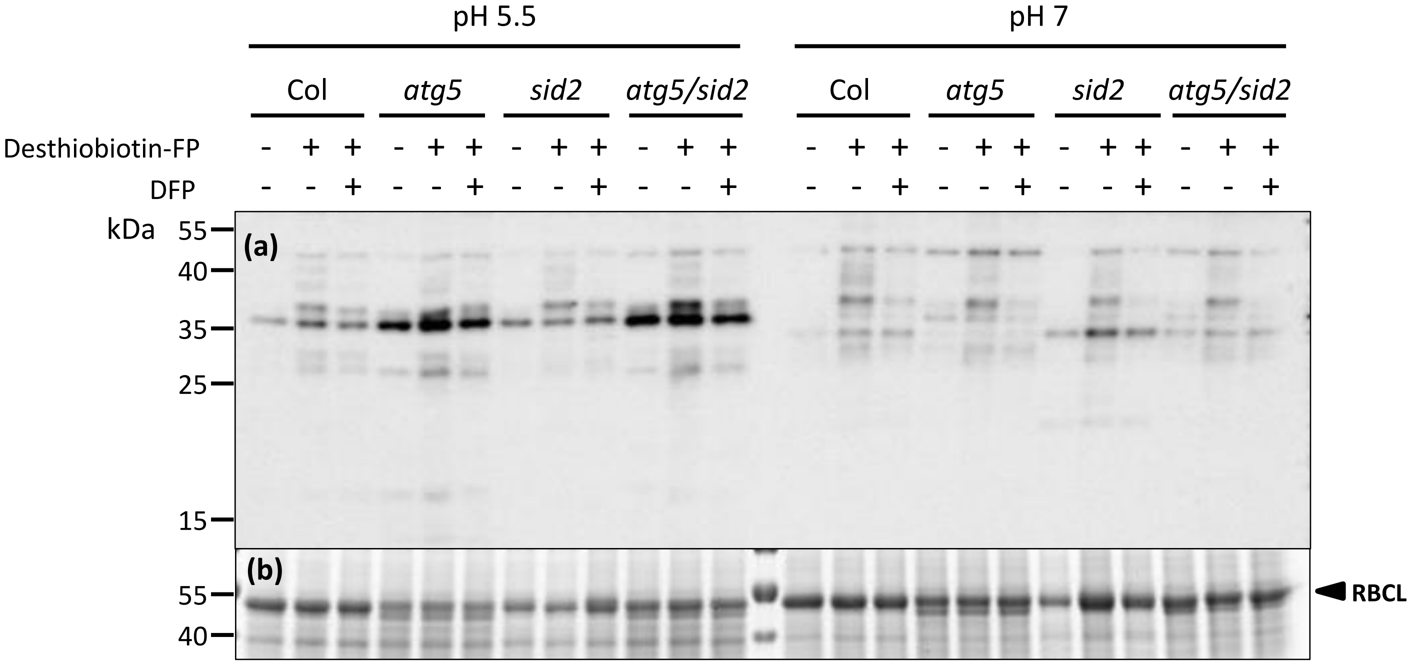

Fig. S7: Desthiobiotin-FP competition assay to estimate the probe's specificity toward serine proteases.

Autophagy defective lines (*atg5* and *atg5*/*sid2*) and their respective controls (Col and *sid2*) were cultivated under high (HN) or low (LN) nitrate conditions for 60 days. Soluble proteins were extracted from rosette leaves using water and protein extracts from HN and LN were pooled at equal protein amount for each genotype. To estimate desthiobiotin-FP specificity toward serine proteases, samples were labelled with desthiobiotin-FP or pre-treated with DFP then labelled with desthiobiotin-FP (competition assay) or simply incubated with the probe's solvent (DMSO) as a no probe control (NPC). As serine proteases are in both vacuole and chloroplast, labelling was performed at both pH 5.5 and 7.5. Serine protease activities (a) were detected using streptavidin-HRP chemiluminescence after separating 10 µg of proteins by SDS-PAGE. (b) The same SDS-PAGE gel was stained with Coomassie brilliant blue (CBB) to check for equal protein input. Rubisco large subunit (RBCL) is indicated by arrow. A band around 35 kDa is detected in the NPC suggesting the presence of an endogenously biotinylated protein. Labelling with desthiobiotin-FP allow the detection of bands between 15 and 55 kDa. These signals were only slightly repressed in the competition assay.

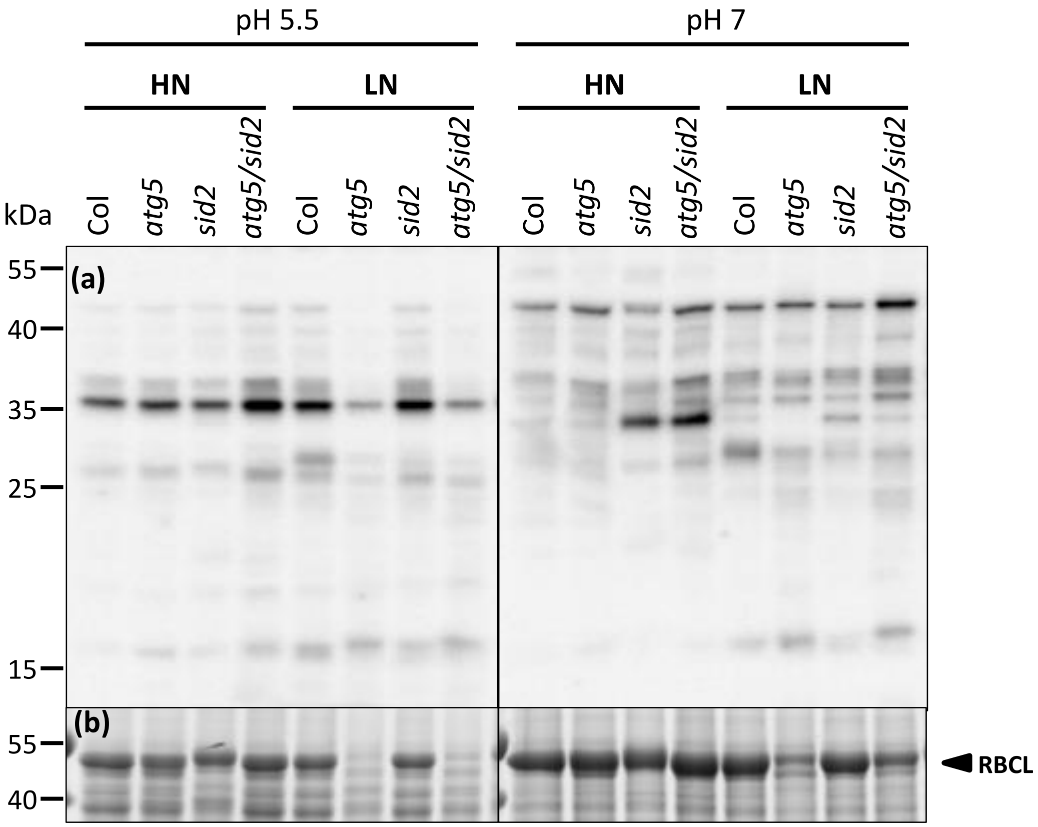

Fig. S8: Serine protease activities are stable in autophagy mutants

Autophagy defective lines (*atg5* and *atg5*/*sid2*) and their respective controls (Col and *sid2*) were cultivated under high (HN) or low (LN) nitrate conditions for 60 days. Soluble proteins were extracted from rosette leaves using water and labelled with desthiobiotin-FP. As serine proteases are localized in both vacuole and chloroplast, labelling was performed at both pH 5.5 and 7.5. Active serine proteasess (a) were detected using streptavidin-HRP chemiluminescence after separating 10 μg of proteins in SDS-PAGE gels and western blot. (b) The same total amount of input proteins was loaded on each lane after incubation as shown by the Coomassie brilliant blue-stained (CBB) protein gel. Rubisco large subunit (RBCL) is indicated by arrow. The pattern of band revealed differs between the two labelling pH suggesting that different serine proteases were actives at acidic or neutral pH. The overall signal was stable between autophagy defective lines and their respective controls at both acidic and neutral pH showing that autophagy deficiency did not induce an increase of active serine proteases.

Table S1: Spectral counts for PLCP peptides in Col, *sid2*, *atg5* and *atg5*/*sid2* according to Fig. 6 (a). Two independent biological replicates from plants grown under low nitrate conditions were analysed (rep 1 and rep 2). Band numbers as shown in Fig. 6 (a) are written in column 2.

		RD21A	SAG12	AALP	CATHB3	RD21B	RD19A	RD19C	CEP1	CATHB2	XCP1
Sample	Band number	AT1G47128	AT5G45890	AT5G60360	AT4G01610	AT5G43060	AT4G39090	AT4G16190	AT5G50260	AT1G02305	AT4G35350
Col_rep 1	1	1	0	0	0	0	0	0	0	0	0
Col_rep 1	2	2	0	0	0	0	0	0	0	0	0
Col_rep 1	3	2	0	0	0	0	0	0	0	0	0
Col_rep 1	4	0	0	0	0	0	0	0	0	0	0
Col_rep 1	5	0	0	1	0	0	0	0	0	0	0
Col_rep 2	1	9	0	0	0	0	0	0	0	0	0
Col_rep 2	2	0	0	0	0	0	0	0	0	0	0
Col_rep 2	3	0	0	0	1	0	0	0	0	0	0
Col_rep 2	4	0	0	0	0	0	0	0	0	0	0
Col_rep 2	5	1	0	0	0	0	0	0	0	0	0
atg5_rep 1	1	24	0	0	0	0	0	0	0	0	0
atg5_rep 1	2	14	0	0	0	3	0	0	0	0	0
atg5_rep 1	3	18	7	0	7	0	5	3	1	4	2
atg5_rep 1	4	11	25	10	0	0	0	0	0	0	0
atg5_rep 1	5	9	8	4	0	0	0	0	0	0	0
atg5_rep 2	1	38	0	0	0	2	0	0	0	0	0
atg5_rep 2	2	15	2	0	0	2	0	0	0	0	0
atg5_rep 2	3	14	7	0	6	0	4	1	1	0	0
atg5_rep 2	4	10	25	15	1	0	0	0	0	0	0
atg5_rep 2	5	5	3	0	0	0	0	0	0	0	0
sid2_rep 1	1	13	0	0	0	0	0	0	0	0	0
sid2_rep 1	2	4	0	0	0	0	0	0	0	0	0
sid2_rep 1	3	0	0	0	0	0	0	0	0	0	0
sid2_rep 1	4	3	0	3	0	0	0	0	0	0	0
sid2_rep 1	5	0	0	3	0	0	0	0	0	0	0
sid2_rep 2	1	17	0	0	0	0	0	0	1	0	0
sid2_rep 2	2	10	0	0	0	0	0	0	0	0	0
sid2_rep 2	3	5	0	0	1	1	0	2	0	0	0
sid2_rep 2	4	5	0	6	0	0	0	0	0	0	0
sid2_rep 2	5	0	0	0	0	0	0	0	0	0	0
atg5/sid2_rep 1	1	60	1	0	0	0	0	0	0	0	0
atg5/sid2_rep 1	2	26	2	0	0	5	0	0	0	0	0
atg5/sid2_rep 1	3	18	8	0	8	0	5	2	2	0	1
atg5/sid2_rep 1	4	8	55	13	0	0	0	0	0	0	0
atg5/sid2_rep 1	5	8	10	5	0	0	0	0	0	0	0
atg5/sid2_rep 2	1	23	0	0	0	0	0	0	0	0	0
atg5/sid2_rep 2	2	15	0	0	0	1	0	0	0	0	0
atg5/sid2_rep 2	3	11	1	0	2	0	0	1	0	0	0
atg5/sid2_rep 2	4	5	16	3	0	1	0	0	0	0	0
atg5/sid2_rep 2	5	2	8	0	0	0	0	0	0	0	0

Tab. S2: Senescence up- and down-effects on the transcription of protease and protease inhibitor genes according to Breeze *et al.*, (2011) transcriptomic data. Breeze *et al*., (2011) classified genes in different clusters (cluster numbers are indicated in column 3) according to their expression patterns. up-regulated during leaf senescence do not present the same pattern of expression. Up-regulated genes during senescence are in classes 27 to 47 (in yellow) while senescence repressed genes are in classes 1 to 26 (presented in green). #N/A indicates that genes were not differentially expressed during leaf ageing. [Breeze *et al*., 2011 High-Resolution Temporal Profiling of Transcripts during Arabidopsis Leaf Senescence Reveals a Distinct Chronology of Processes and Regulation. *Plant Cell* **23**(3): 873-894.]

AGI	Name	Senescence effect
AT4G38220	AQI	#N/A
AT2G27020	PAG1	#N/A
AT2G24200	LAP1	#N/A
AT4G20850	TPP2	#N/A
AT5G35590	PAA1	#N/A
AT4G31300	PBA1	#N/A
AT3G22110	PAC1	#N/A
AT2G05840	PAA2	#N/A
AT4G14800	PBD2	#N/A
AT3G60820	PBF1	#N/A
AT1G53750	RPT1A	#N/A
AT3G05530	RPT5A	#N/A
AT4G17510	UCH3	#N/A
AT5G05780	RPN8A	#N/A
AT5G58290	RPT3	#N/A
AT5G10540	TOP2	2
AT1G21720	PBC1	#N/A
AT1G56450	PBG1	#N/A
AT5G42790	PAF1	#N/A
AT4G01610	CTB3	46
AT1G47128	RD21A	46
AT1G53850	PAE1	#N/A
AT5G45890	SAG12	40
AT5G51070	CLPD	#N/A
AT3G13235	DDI1	#N/A
AT1G79340	AMC4	#N/A
AT1G50380	_	#N/A
AT3G51260	PAD1	#N/A
AT5G23540	RPN11	#N/A
AT4G38630	RPN10	#N/A
AT1G51710	UBP6	#N/A
AT5G66140	PAD2	#N/A
AT4G30910	LAP3	#N/A
AT4G39090	RD19A	31
AT5G43060	RD21B	44
AT5G10760	AED1	#N/A
AT1G16470	PAB1	#N/A
AT1G79210	PAB2	#N/A
AT2G14260	PIP	#N/A
AT5G23140	CLPP2	#N/A
AT4G16190	RD19C	47
AT5G26860	LON1	#N/A
AT3G14290	PAE2	#N/A
AT3G20630	UBP14	#N/A
AT2G41790	PXM16	#N/A
AT4G36760	APP1	#N/A
AT5G36210	_	#N/A
AT5G50260	CEP1	43
AT1G02305	CTB2	47
AT4G35350	XCP1	22
AT5G60360	AALP	31
AT1G50250	FTSH1	26
AT5G42270	FTSH5	25
AT4G36195	_	31
AT1G09750	AED3	19
AT3G54400	_	24
AT3G02110	SCPL25	22
AT2G39850	SBT4.1	#N/A
AT1G52510	_	5
AT2G47940	DEGP2	18
AT2G35780	SCPL26	20
AT5G39830	DEGP8	16
AT3G27925	DEGP1	#N/A
AT3G14067	SASP	36
AT3G19170	PREP1	#N/A
AT5G42390	SPP	#N/A
AT3G24590	PLSP1	8
AT1G13270	MAP1C	10
AT5G05740	EGY2	17
AT2G30950	FTSH2	21
AT4G18370	DEGP5	17
AT3G52500	_	19
AT5G42240	SCPL42	#N/A
AT1G06430	FTSH8	#N/A
AT5G23210	SCPL34	#N/A
AT3G05350	_	#N/A
AT5G65760	_	28
AT1G67700	HHL1	#N/A
AT3G61820	_	19
AT1G01300	APF2	25
AT4G34980	SLP2	7
AT2G05920	SBT1.8	14
AT3G18490	ASPG1	#N/A
AT2G33530	SCPL46	#N/A
AT4G21650	SBT3.13	13
AT5G08260	SCPL35	18
AT1G09130	CLPR3	#N/A
At1g21750	PDIL1-1/PDI5	#N/A
At1g47710	Serpin-1	#N/A
At4g16500	CYS4	#N/A
At3g12490	CYS6	33
At5g12140	CYS1	#N/A
