# Supplementary material for: Increases in activity of proteasome and papain-like cysteine protease in Arabidopsis autophagy mutants: back-up compensatory effect or cell-death promoting effect?
Source: J Exp Bot. 2018 Jan 27;69(6):1369–85. doi: 10.1093/jxb/erx482 (PMC6037082; doi:10.1093/jxb/erx482)
Supplement: Supplementary Methods [file erx482_suppl_supplementary_methods.pdf]

Increase of proteasome and papain-like cysteine protease activities in autophagy mutants: backup compensatory effect of pro cell-death effect?

Marien Havé[1], Thierry Balliau[2], Betty Cottyn-Boitte[1], Emeline Dérond[1], Gwendal Cueff[1], Fabienne Soulay[1], Aurélia Lornac[3], Pavel Reichman[4], Nico Dissmeyer[4], Jean-Christophe Avice[3], Patrick Gallois[5], Loïc Rajjou[1], Michel Zivy[2], Céline Masclaux-Daubresse[1*]

Supplementary Method 1: Phenotyping display and growth conditions

The production of homogeneous vegetative plant material was performed in controlled conditions (growth chamber). Two N environments were compared at the same time and in the same growth chamber in each experiment (cultivation repetition). Plants were grown in small pots (L = 60 mm, l = 65 mm, h = 60 mm) containing six plants positioned on a circle for low nitrate conditions and containing one plant per pot for high nitrate conditions. The two N environments compared in this study have been used previously in all our N-related studies (Diaz *et al.*, 2005; 2006; 2008; Lothier *et al.*, 2011; Guiboileau *et al.*, 2013; Masclaux-Daubresse *et al.*, 2014). The high nitrate condition did not limit plant growth at any stage during our experiment and the second one (low nitrate) strongly limited growth. Pots were filled with sand and watered (by immersion of the base of the pots) in a solution containing either 10 mm (HN) or 2 mm (LN) nitrate. Phosphate and sulfate were present in both solutions at the same concentration (0.25 mm), as well as magnesium (0.25 mm) and sodium ions (0.20 mm). The difference between HN and LN solutions concerned only potassium (5.25 mm and 2.75 mm, respectively, in HN and LN solutions), calcium (2.50 mm and 0.50 mm, respectively), and chloride ions (0.20 mm and 0.70 mm, respectively), but all these concentrations were supraoptimal for plant growth. HN and LN solutions contained desirable amounts of Fe and other micronutrients. The pH of the watering solutions remained between 5.1 and 5.5.

References:

1. Diaz C, Lemaitre T, Christ A, Azzopardi M, Kato Y, Sato F, Morot-Gaudry JF, Le Dily F, Masclaux-Daubresse C. 2008. Nitrogen recycling and remobilization are differentially controlled by leaf senescence and development stage in Arabidopsis under low nitrogen nutrition. Plant Physiology 147, 1437-1449.
2. Diaz C, Purdy S, Christ A, Morot-Gaudry J-F, Wingler A, Masclaux-Daubresse C. 2005. Characterization of markers to determine the extent and variability of leaf senescence in Arabidopsis. A metabolic profiling approach. Plant Physiol 138, 898-908.
3. Diaz C, Saliba-Colombani V, Loudet O, Belluomo P, Moreau L, Daniel-Vedele F, Morot-Gaudry J-F, Masclaux-Daubresse C. 2006. Leaf yellowing and anthocyanin accumulation are two genetically independent strategies in response to nitrogen limitation in Arabidopsis thaliana. Plant Cell Physiol 47, 74-83.
4. Guiboileau A, Avila-Ospina L, Yoshimoto K, Soulay F, Azzopardi M, Marmagne A, Lothier J, Masclaux-Daubresse C. 2013. Physiological and metabolic consequences of autophagy defisciency for the management of nitrogen and protein resources in Arabidopsis leaves depending on nitrate availability. New Phytologist 199, 683-694.

5. Lemaitre T, Gaufichon L, Boutet-Mercey S, Christ A, Masclaux-Daubresse C. 2008. Enzymatic and metabolic diagnostic of nitrogen deficiency in Arabidopsis thaliana Wassileskija accession. Plant and Cell Physiology 49, 1056-1065.
6. Lothier J, Gaufichon L, Sormani R, Lemaitre T, Azzopardi M, Morin H, Chardon F, Reisdorf-Cren M, Avice J-C, Masclaux-Daubresse C. 2011. The cytosolic glutamine synthetase GLN1;2 plays a role in the control of plant growth and ammonium homeostasis in Arabidopsis rosettes when nitrate supply is not limiting. Journal of Experimental Botany 62, 1375-1390.
7. Masclaux-Daubresse C, Clément G, Anne P, Routaboul J, Guiboileau A, Soulay F, Shirasu K, Yoshimoto K. 2014. Stitching together the multiple dimensions of autophagy using metabolomic and transcriptomic analyses reveals new impacts of autophagy defects on metabolism, development and plant response to environment. The Plant Cell 26, 1857-1877.

Supplementary Method 2: Shotgun proteomic analysis

Sample preparation for shotgun proteomics.

Rosette leaves were ground and reduced to a fine powder using liquid nitrogen in a mortar. Three biological replicates from each genotype and nutrition treatment were prepared for the shotgun LC-MS/MS analysis. Leaf total proteins were extracted using a TCA-acetone method (Méchin *et al.*, 2007). Briefly, 100 mg of finely ground leaf powder were homogenized in 1.4 ml of cold precipitating solution (10% TCA, 0.07% β-mercaptoethanol in acetone) and incubated at -20°C for 90 min. After centrifugation (20 000 x *g* at 40°C, 15 min), the pellets were washed three times with cold acetone containing 0.07% β-mercaptoethanol and dried using a speedvacuum system. Pellets were solubilized in 100 μl of ZUT buffer containing 6 M urea, 2 M thiourea, 10 mM DTT, 30 mM Tris-HCl pH 8.8 and 0.1% ZALS (zwitterionic acid labile surfactant, Proteabio, Morgantown, WV, USA) and homogenized for 3 min with a vortex. After centrifugation (14 000 x g at 25°C, 30 min), protein concentrations in the supernatant were assayed using the 2D Quant kit (GE healthcare) using BSA as a standard and following supplier's instructions.

For each sample, 10 μl of a 4 $\mu g.\mu l^{-1}$ protein solution was equilibrated for 30 min at room temperature. Proteins were alkylated by incubation in darkness for 1 h at room temperature after addition of 2 μl of 330 mM Iodoacetamide (in 50 mM ammonium bicarbonate). Proteins were then diluted ten times by addition of 90 μl of 50 mM ammonium bicarbonate and digested by addition of 4 μl of a trypsin solution (0.2 $g.\mu l^{-1}$ in 50 mM acetic acid) at a 50:1 ratio (soluble proteins/trypsin) and incubation overnight at 37°C. Digestion was stopped by addition of 6 μl of 18.6% trifluoroacetic acid (TFA, final concentration of 1%). Digested proteins were desalted using a Strata^{TM}-XL polymeric reversed phase column (100 μm, Phenomex, Le Pecq, France) according to Duruflé *et al.*, (2017) and solubilized with 2% acetonitrile (ACN) and 0.8% formic acid (FA).

LC-MS/MS analysis

HPLC was performed on a Nano-HPLC (Eksigent). Buffers A and B were prepared with 0.1% FA in water, and with 0.1% FA in ACN, respectively. A 4 μL sample of the peptide solution was loaded at 7.5 $\mu L\ min^{-1}$ for 1 min on a Biosphere C18 trap-column (particle size:

5 µm, pore size: 12 nm, inner/outer diameters: 360/100 µm, length: 20 mm; NanoSeparations) and desalted with 0.1% FA, 2% ACN in water. Then peptides were separated on a biosphere C18 column (particle size: 3 µm, pore size: 12 nm, inner/outer diameters: 360/75 µm, length: 300 mm; NanoSeparations). The peptide separation was achieved at 300 nl min^{-1} with the following steps: equilibration in 95% buffer A for 9 min, separation with a linear gradient from 5 to 30% of buffer B for 110 min, linear gradient from 35 to 95% buffer B for 3 min and regeneration with 95% buffer B for 10 min.

Eluted peptides were on-line analyzed with a Q-Exactive mass spectrometer (Thermo Fisher Scientific) using a nanoelectrospray interface. Ionization (1.5 kV ionization potential) was performed with a glass needle (non-coated capillary silica tips, 360/20-10, New Objective). Peptide ions were analyzed using Xcalibur 2.07 with the following data-dependent acquisition steps: (1) full MS scan (mass-to-charge ratio [m/ z], 400 to 1 400; profile mode) with a resolution of 70 000 and (2) MS/MS (isolation window = 3 m/z, AGC target = 5e4, max Ion Time = 120 ms, collision energy = 27%; profile mode, resolution = 17 500). Step 2 was repeated for the eight major ions detected in step 1. Dynamic exclusion was set to 40 s. Only the doubly and triply charged precursor ions were subjected to MS/MS fragmentation.

Xcalibur raw data were transformed to mzXML open source format and centroided using the msconvert software in the ProteoWizard 3.0.3706 package (Kessner *et al*,. 2008). Protein identification was performed using the X!Tandem Piledriver (version 2015.04.01; www.thegpm.org) by querying MS/MS data against the TAIR10 protein library together with a custom contaminant database (trypsin, keratins). Following parameters were used: one missed trypsin cleavage allowed, alkylation of cysteine and oxidation of methionine were set to static and possible modification, respectively. Precursor mass tolerance was set to 10 ppm and fragment ion mass tolerance was 0.02 Da. A refinement search was added with similar parameters except that the missed cleavage was set to three and possible N-terminal acetylation with peptide signal cleavage was searched. Identified proteins were filtered and grouped using X!Tandem Pipeline (3.4.1) (pappso.inra.fr/bioinfo/xtandempipeline/) (Langella *et al*., 2017) according to: (1) a minimum of two different peptides required with an E value smaller than 0.01, (2) a protein E value (calculated as the product of unique peptide E values) smaller than 10^{-5}. The false discovery rates (FDRs) at peptide and protein level were 0.03% and 0.0%, respectively.

Relative quantification was performed using the MassChroQ software (pappso.inra.fr/bioinfo/masschroq/) (Valot *et al*., 2011) by peak area integration on extracted ion chromatograms (XICs) within a 10 ppm window, after LC-MS/MS chromatogram alignment and spike filtering. Data were than filtered for shared, unreproducible and uncorrelated peptides. The peptides shared by two or more proteins were removed. Peptides which were quantified in less than 5% of the sample were considered unreproducible and removed. Finally, peptides whose intensity profile deviate from the average profile of the peptides belonging to the same protein (with a coefficient of correlation inferior to 0.7) were removed. Relative protein abundance was thus calculated and defined as the sum of XICs intensities of (1) reproducible peptides, (2) specific peptides and (3) correlated peptides belonging to a same protein. When the peptides of a protein were not present or not

reproducibly observed in one or several conditions, spectral counting (SC) was used in place of XICs analysis.

References:

Durufle H, San Clemente H, Balliau T, Zivy M, Dunand C, Jamet E. 2017. Cell wall proteome analysis of Arabidopsis thaliana mature stems. Proteomics 17(8). doi: 10.1002/pmic.201600449

Kessner D, Chambers M, Burke R, Agusand D, Mallick P. 2008. ProteoWizard: open source software for rapid proteomics tools development. *Bioinformatics* **24**: 2534-2536.

Langella O, Valot B, Balliau T, Blein-Nicolas M, Bonhonarne L, Zivy M. 2017. X!TandemPipeline: A Tool to Manage Sequence Redundancy for Protein Inference and Phosphosite Identification. *Journal of Proteome Research* **16**: 494-503.

Méchin V, Damerval C, Zivy M 2007. Total Protein Extraction with TCA-Acetone. In: Thiellement H, Zivy M, Damerval C, Méchin Vr eds. *Plant Proteomics: Methods and Protocols*. Totowa, NJ: Humana Press, 1-8.

Valot B, Langella O, Nano E, Zivy M. 2011. MassChroQ: A versatile tool for mass spectrometry quantification. *Proteomics* **11**: 3572-3577.

Supplementary Method 3: Shotgun LC-MS/MS analysis of pulled-down PLCPs

In-gel digestion of the excised bands was performed with the Progest system (Digilab, Proteomic Solutions). First, the gel pieces were washed following this procedure: 15 min in 40% ethanol/10% acetic acid, 15 min in ACN, 15 min in 25 mM ammonium bicarbonate and 15 min in 100% ACN. The last two steps were repeated twice then the gel pieces were incubated 10 min in 100% ACN. Proteins were reduced in the presence of 10 mM DTT in 25 mM ammonium bicarbonate for 30 min at 58°C. Alkylation was performed by incubation with 55 mM iodoacetamide in 25 mM ammonium bicarbonate for 40 min at room temperature in the dark. Gel pieces were then incubated 15 min in 25 mM of ammonium bicarbonate followed by 15 min incubation in 100% ACN. The previous two steps were repeated twice. Gel plugs were then washed with 100% ACN and dried. Digestion was performed for 7 h at 37°C with 125 ng of trypsin (Promega) dissolved in 20% methanol and 25 mM ammonium bicarbonate. Trypsic peptides were extracted with 50% ACN and 0.5% TFA and then with 100% ACN. Peptide extracts were dried using a speedvacuum system and suspended in 30 μl of 2% ACN and 0.08% FA.

HPLC was performed on an Ultimate 3000 RSLCnano (ThermoFinnigan). Buffers A and B were prepared with 2% ACN, 0.1% TFA and with 80% ACN, 0.1% FA, respectively. A 4 µL sample of the peptide solution was loaded at 20 µL min^{-1} for 5 min on a PepMap 100 C18 (particle size: 5 µm, pore size: 100 Å, diameters: 300 µm, length: 5 mm). Then peptides were separated on an Acclaim PepMap RSLC (particle size: 2 μm, pore size: 100 Å, diameters: 75 μm, length: 15 cm; nanoViper). The peptide separation was achieved with the following steps: linear gradient from 4 to 39% of buffer B for 14 min, linear gradient from 35 to 99% of

buffer B for 30 s. Then, 99% of buffer B for 90 s, linear gradient from 99 to 2% of buffer B for 30 s and then 2% of buffer B for 3 min and 30 s.

Eluted peptides were on-line analyzed with a LTQ-Orbitrap discovery (ThermoFinnigan) using a nanoelectrospray interface. Ionization was performed with liquid junction at 1.4 kV. Peptide ions were analyzed using Xcalibur 2.07 with the following data-dependent acquisition steps: (1) full MS scan (mass-to-charge ratio [m/ z], 300 to 1 400; centroid mode) with a resolution of 15 000 and (2) MS/MS on the six major ions detected in the LTQ trap with a CID collision mode (activation Q = 0,220, activation time = 50 ms, collision energy = 35%; centroid mode). Dynamic exclusion was set to 30 s. Only the doubly and triply charged precursor ions were subjected to MS/MS fragmentation.

Protein identification was performed as described above using X!Tandem Piledriver. Identified proteins were filtered and grouped using X!Tandem Pipeline (3.4.2) according to: (1) ~~A~~ a minimum of two different peptides required with an E value smaller than 0.05, (2) a protein E value (calculated as the product of unique peptide E values) smaller than 10^{-4}.
